# TCR–pMHC bond conformation controls TCR ligand discrimination

**DOI:** 10.1038/s41423-019-0273-6

**Published:** 2019-09-17

**Authors:** Dibyendu K. Sasmal, Wei Feng, Sobhan Roy, Peter Leung, Yanran He, Chufan Cai, Guoshuai Cao, Huada Lian, Jian Qin, Enfu Hui, Hans Schreiber, Erin J. Adams, Jun Huang

**Affiliations:** 1 The Pritzker School of Molecular Engineering, The University of Chicago, Chicago, IL USA; 2 Department of Biochemistry and Molecular Biology, The University of Chicago, Chicago, IL USA; 3 Department of Pathology, The University of Chicago, Chicago, IL USA; 4 Department of Materials Science & Engineering, Stanford University, Stanford, CA USA; 5 Department of Chemical Engineering, Stanford University, Stanford, CA USA; 6 Section of Cell & Developmental Biology, Division of Biological Sciences, University of California, San Diego, CA USA

**Keywords:** Bond conformation, T cell receptor, Single molecule FRET, ligand discrimination, MHC class II, Cellular immunity

## Abstract

A major unanswered question is how a TCR discriminates between foreign and self-peptides presented on the APC surface. Here, we used in situ fluorescence resonance energy transfer (FRET) to measure the distances of single TCR–pMHC bonds and the conformations of individual TCR–CD3ζ receptors at the membranes of live primary T cells. We found that a TCR discriminates between closely related peptides by forming single TCR–pMHC bonds with different conformations, and the most potent pMHC forms the shortest bond. The bond conformation is an intrinsic property that is independent of the binding affinity and kinetics, TCR microcluster formation, and CD4 binding. The bond conformation dictates the degree of CD3ζ dissociation from the inner leaflet of the plasma membrane via a positive calcium signaling feedback loop to precisely control the accessibility of CD3ζ ITAMs for phosphorylation. Our data revealed the mechanism by which a TCR deciphers the structural differences among peptides via the TCR–pMHC bond conformation.

## Introduction

A TCR consists of a variable TCRαβ heterodimer and a nonvariable transmembrane signal transduction CD3 complex containing CD3γε and CD3δε heterodimers and a CD3ζζ homodimer. TCRs specifically and sensitively detect a small number of agonist pMHCs among a plethora of structurally similar self-pMHCs to trigger antigen-specific immune responses.^1–3^ Despite intense efforts, the mechanism underlying TCR ligand discrimination remains a major unanswered question in immunology.^4–6^ TCR ligand discrimination is uniquely challenging. First, it requires TCRs to detect very rare foreign pMHCs in the presence of considerably abundant self-pMHCs. Second, it requires proper signaling propagation from surface TCR binding to induce intracellular CD3 phosphorylation. Although it is generally thought that the engagement of the extracellular TCRαβ domain with a pMHC results in biochemical changes in the cytoplasmic portions of the CD3 complex, there are no experimental data that can directly depict this process with enough spatiotemporal resolution at the membranes of live primary T cells.

Many models have been proposed to explain the molecular mechanism underlying TCR discrimination. The TCR conformational change model postulates that a conformational change in a TCR occurs upon pMHC binding, but no conformational changes at the TCR–pMHC binding interface have been identified that are conserved in TCR–pMHC crystal structures.^5,7^ However, crystal structures only provide a “snapshot” of the thermodynamically stable conformations of purified TCR and pMHC proteins. The TCR is an active molecular machine that is anchored at the live cell membrane, associates with CD3 signaling units, links to the cytoskeleton, and interacts with other signaling molecules. “Active” TCR molecules at cell membrane are very different from purified “quiescent” TCR proteins isolated from the cellular environment. A typical example of this is that the in situ binding kinetics and affinities of TCR–pMHC interactions measured at the T-cell membrane are dramatically different from those measured in vitro in solution.^3,8^ It has long been speculated that the TCR at the cell membrane undergoes conformational changes upon pMHC binding. This hypothesis is attractive, but it has never been experimentally proven at the membranes of live primary T cells, mainly due to the lack of appropriate experimental approaches. Furthermore, another key issue for TCR discrimination is how different TCR–pMHC interactions result in distinct biochemical changes in the cytoplasmic domains of CD3. It has been suggested that CD3 ITAMs are sequestered in the plasma membrane and TCR–pMHC engagement pulls the CD3 cytoplasmic domains away from the membrane, thus making the ITAMs accessible to phosphorylation by Lck.^9^ However, other studies argue that the release of ITAMs from the plasma membrane is unlikely to be a prerequisite step in the initiation of TCR signaling.^6^

To carefully examine possible TCR conformational changes and investigate the molecular mechanism of TCR triggering in situ, we used fluorescence resonance energy transfer (FRET),^10,11^ which functions as a spectroscopic ruler with subnanometer precision, to measure the intermolecular distance of a TCR–pMHC bond and the intramolecular conformation of a TCR–CD3ζ complex at the immunological synapse of a live primary CD4^+^ T cell in real time with high spatiotemporal resolution. These experiments enabled us to critically test the TCR conformational change model and probe the molecular mechanism underlying TCR ligand discrimination.

## Results

### FRET design

**Fig. 1:**
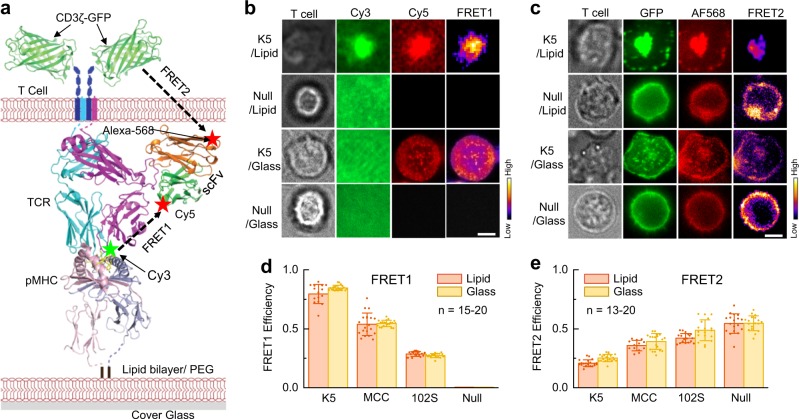
Measurement of TCR conformational dynamics by FRET. **a** A composite structural model of 5C.C7 TCR (PDB ID: 4P2R), MCC-IE^k^ (PDB ID: 3QIU), scFv (PDB ID: 1NFD), and CD3ζ–GFP (PDB ID for GFP: 1GFL). To measure the TCR–pMHC bond conformational dynamics using FRET1, the TCR was labeled by Cy5 via the scFv J1, and the peptide C-terminus was labeled by Cy3. For determining the TCR–CD3ζ conformational changes by FRET2, the TCR was labeled by Alexa Fluor 568 (Alexa568) via scFv J3, and the CD3ζ C-terminus was tagged with GFP. Extracellular Cy3/Cy5 FRET1 and transmembrane GFP/Alexa568 FRET2 are indicated by dashed lines. The pMHC molecules were anchored on either a lipid bilayer or on a PEG-Ni^2+^ glass surface (Fig. S1a). Note: J1 and J3 are different scFvs, and each has only one unique labeling site. **b**, **c** T cell, donor, acceptor, and calculated FRET signals of TCRs interacting with K5 or a null pMHC on a lipid bilayer or on a glass surface. The calculated FRET efficiency images for FRET1 (**b**) and FRET2 (**c**) are shown in pseudocolor; the cold-to-hot color spectrum represents weak-to-strong FRET efficiency. Representative data from 3–5 independent experiments for each pMHC at 37 °C are shown. The scale bar is 5 µm. **d**, **e** FRET efficiencies measured for K5, MCC, 102S, and null pMHCs on a lipid bilayer (red) and a glass surface (yellow) for extracellular TCR–pMHC FRET1 (**d**) and transmembrane TCR–CD3ζ FRET2 (**e**). At least 13 cells were used to determine the FRET efficiency for each pMHC. Also see Figs. S1–4 and Movies S1–3

To determine the conformation of a single TCR–pMHC bond, a TCR and a pMHC were site-specifically labeled with the FRET acceptor Cy5 and the FRET donor Cy3, respectively.^8^ The peptide within the MHC molecule was labeled with Cy3, and the TCR was labeled with the anti-TCR single-chain variable fragment (scFv) J1-Cy5 (Fig. 1a and Fig. S1a). The intermolecular distance between Cy3 and Cy5 provided a reasonable approximation of the conformation (or compactness) of the TCR–pMHC bond. The conformation of a single TCR–pMHC bond on the cell surface was measured by Cy3/Cy5 FRET (FRET1) (Fig. 1a) in real time by total internal reflection fluorescence (TIRF) microscopy (Fig. S1b). The Cy3/Cy5 inter-dye distance of a TCR–pMHC bond is denoted as the TCR–pMHC bond distance to describe the TCR–pMHC bond conformation in the following paragraphs. To measure the intramolecular distance between the TCR and CD3ζ in a transmembrane TCR–CD3ζ complex, we added a green fluorescent protein (GFP) to the C-terminus of the CD3ζ chain, and the TCR was labeled with an Alexa Fluor 568 fluorophore using a different anti-TCR scFv J3 with a unique labeling site close to the cell membrane.^8^ The real-time intramolecular distances of the TCR–CD3ζ complexes were measured by GFP/Alexa Fluor 568 FRET (FRET2) (Fig. 1a) using epifluorescence time-lapse microscopy (Fig. S1b).

We first performed experiments to assess the feasibility and specificity of cell surface FRET1 and transmembrane FRET2 on a lipid bilayer (Fig. 1a) and on a glass surface (Fig. S1a) containing the pMHC and accessory molecules ICAM-1 and B7-1, respectively (Fig. S2 and Movie S1–3). For cell surface FRET1, we readily detected FRET signals from three agonist pMHCs but not from a null pMHC on the lipid bilayer and on the glass surface, and the FRET efficiencies (*E*_FRET_) were positively correlated with the pMHC potencies in activating T cells in vitro^12^ (Fig. 1b, d). The average synaptic *E*_FRET1_ was 0.79, 0.54, and 0.29 for the super agonist K5, the agonist MCC, and the weak agonist 102S, respectively. However, no synaptic FRET was observed for the null pMHC. These data validated the specificity of the cell surface TCR–pMHC FRET and were consistent with the results of a previous report.^8^ In contrast, the transmembrane TCR–CD3ζ FRET2 efficiencies were inversely correlated with the pMHC potencies, and the highest FRET was observed for the null pMHC (Fig. 1c, e). In the presence of the K5, MCC, and 102S pMHCs, the transmembrane FRET change was only detected in the TCR–CD3ζ colocalized microclusters and not outside of the colocalized microclusters (Fig. 1c and Fig. S3a). Replacing the FRET acceptor Alexa Fluor 568 with a Cy5 dye abolished the transmembrane FRET2 (Fig. S3b). These experiments confirmed the specificity of the transmembrane TCR–CD3ζ FRET.

### Measurement of the TCR–pMHC bond conformation by smFRET

**Fig. 2:**
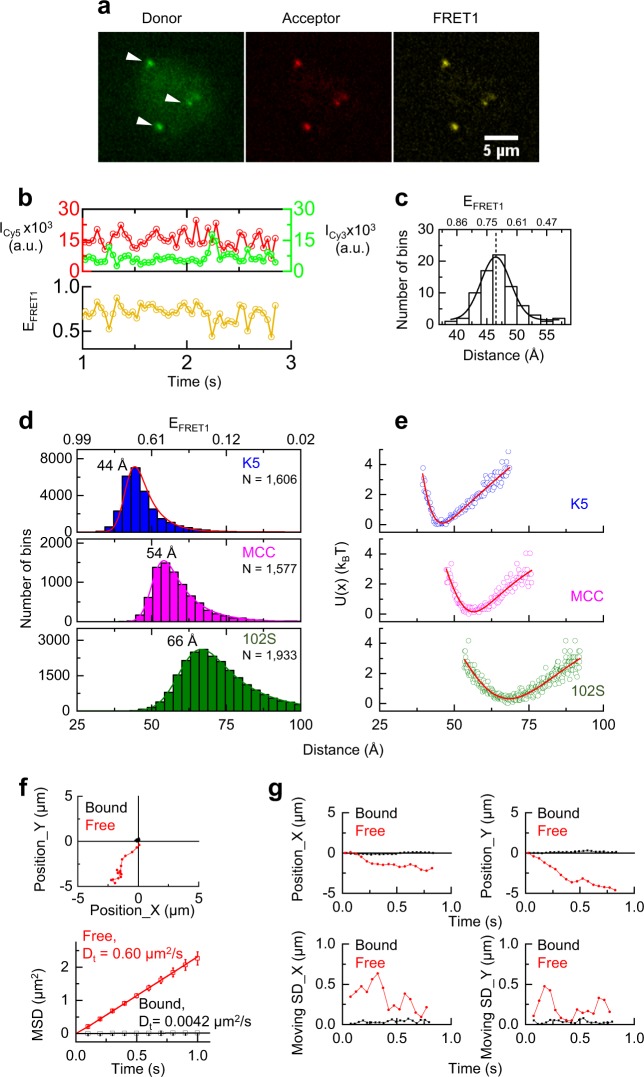
The conformational dynamics of single TCR–pMHC bonds. **a** A representative smFRET event mediated by the interaction between a 5C.C7 TCR and a K5 pMHC. The donor, acceptor, and FRET signals are shown, and white arrows indicate single molecules. **b** Single-molecule time trajectories of the donor (green, Cy3-pMHC) and the acceptor (red, Cy5-TCR) intensities (upper panel) and the corresponding time trajectory of smFRET efficiency (yellow, lower panel). Single donor and acceptor molecules were tracked in real time to calculate the FRET efficiency. Also see Fig. S5. **c** Histogram of the Cy3–Cy5 distances that was calculated from the smFRET efficiencies shown **b** (lower panel, see methods) and fitted according to a Gaussian distribution (black curve). Also see Fig. S6. **d** Histograms of the TCR–pMHC bond distances for a single bond for the K5, MCC, and 102S pMHCs. Each histogram used 1577–1933 trajectories to determine the most probale TCR–pMHC bond distance for each pMHC. Also see Figs. S7 and S8. **e** PMF determination of single TCR–pMHC bonds for the K5, MCC, and 102S pMHCs. PMF indicates the free energy changes as a function of the TCR–pMHC bond distance. **f** Representative diffusion trajectories of single pMHCs under free (red) and bound (black) conditions (top panel). The mean squared displacement (MSD) vs. time (*t*) was plotted to show the mobility of single-tracked pMHCs (*x*-axis: acquisition time in seconds; *y*-axis: MSD). The calculated diffusion coefficients (*D*_*t*_) were determined for each type of diffusion (bottom panel). **g** The horizontal positions *x* and vertical positions *y* of each single-tracked pMHC were plotted vs. time *t* for representative single free (red) and bound (black) pMHCs (top panels). The corresponding sliding standard deviation σ for three consecutive points according to the *x* and *y* positions were calculated to reveal the position changes of the single-tracked pMHCs (bottom panels)

We next performed Cy3/Cy5 single-molecule FRET (smFRET) on a lipid bilayer to measure the conformations of single TCR–pMHC bonds using TIRF microscopy (Fig. 2 and Fig. S1b). TCR–pMHC bond formation at live T-cell membrane brought the donor Cy3 and the acceptor Cy5 into close enough proximity to produce smFRET (Figs. 1a and 2a). The fluorescent intensities of Cy3 and Cy5 were simultaneously recorded in real time (Fig. 2b, top panel, and Fig. S4a), and the *E*_FRET_ values were calculated based on the fluorescence intensities of the donor Cy3 and the acceptor Cy5 (Fig. 2b, bottom panel). As *E*_FRET_ is inversely proportional to the distance between the donor and the acceptor to the sixth power, Cy3/Cy5 smFRET serves as a sensitive microscopic ruler to precisely measure the intermolecular TCR–pMHC bond distance in real time during binding. The time trajectory of *E*_FRET_ showed that the TCR–pMHC bond is dynamic and exhibits continuous conformational changes (Fig. 2b), which is in line with the results in recent reports that demonstrated that a TCR or a pMHC undergoes conformational changes upon binding.^13,14^ The recording of the fluctuating conformational trajectories provided for the real-time observation of single TCR–pMHC bond dynamics. By plotting the *E*_FRET_ histogram and fitting it with a Gaussian function, we determined the most probale *E*_FRET_ value of 0.7, corresponding to a 47 ± 3 Å distance between the 5C.C7 TCR and the super agonist K5 pMHC in a representative smFRET trajectory (Fig. 2c). We repeated our single-bond measurements for the K5 pMHC and performed smFRET experiments for the agonist MCC and weak agonist 102S pMHCs (Fig. S5). After collecting many individual smFRET trajectories for each pMHC, we pooled all *E*_FRET_ data together and plotted the histograms for each pMHC (1577–1933 trajectories per histogram, Fig. 2d). Remarkably, the distributions of *E*_FRET_ showed that the intermolecular TCR–pMHC bond distances were peptide dependent, and single TCR–pMHC bonds were highly dynamic within a continuous range of conformational states. Fitting each histogram with a Gaussian function (curves, Fig. 2d) yielded the most probable intermolecular TCR–pMHC bond distance for each pMHC: 44 ± 9 Å for K5 (super agonist), 54 ± 11 Å for MCC (agonist), and 66 ± 18 Å for 102S (weak agonist) (Fig. 2d), directly revealing the angstrom-level, binding-induced, peptide-dependent TCR–pMHC bond distances and conformational dynamics in situ. This key information was missing from previously reported TCR–pMHC crystal structures.^7,15^ In addition, we experimentally verified that the differences in the distances of the pMHCs were not due to peptide labeling-derived noise (Fig. S6). We further measured the average TCR–pMHC bond distances using ensemble FRET on both a lipid bilayer and a glass surface (Figs. S4b and S7), and the results confirmed the single-bond measurements obtained using smFRET (Fig. 2d).

We then quantified the binding strength of each single TCR–pMHC bond by analyzing the potential-of-mean-force (PMF),^16^ which measures the free energy cost of the variation in bond conformation. PMF is at minimum when the bond conformation is at equilibrium, and the PMF curve indicates the sizes of the fluctuations (Fig. 2e). Clearly, the super agonist K5 and agonist MCC have deep and narrow energy wells, indicating their strong and stable bonds. In contrast, the weak agonist 102S has a shallow and wide energy well, suggesting its weak bond strength and unstable binding state. Overall, the depth and width of the PMF curve revealed that K5 and MCC form more stable (shorter) bonds with TCRs compared with 102S (Fig. 2e), which is consistent with previous reports that indicated that K5 and MCC have higher 3D in vitro binding affinities with TCRs than 102S^8,12^ and that TCR triggering is dependent on the receptor–ligand complex dimensions.^17,18^ Our smFRET measurements not only revealed that TCR triggering is critically dependent on the conformation of a TCR–pMHC bond but also linked the bond conformation to the bond energy, thus providing a fundamental basis for understanding TCR ligand discrimination.

### TCR–pMHC bond conformation is an intrinsic property independent of binding kinetics and affinity

Cell surface smFRET is highly distance dependent^8^ and only produces a FRET signal when a TCR binds to a pMHC (Fig. 1b, d, comparison of K5 with null). To confirm the bound state, we tracked and compared the diffusion of single pMHCs in smFRET and those of individual-free pMHCs on the lipid bilayer (Fig. 2f, g and Fig. S8). We found that the pMHCs in smFRET were tightly restricted within the synapse, whereas the free pMHCs were very mobile, as shown by their distinct diffusion trajectories and coefficients (Fig. 2f, top panel). The diffusion coefficient of a pMHC in smFRET was close to 0, which was 140-fold smaller than that of a free pMHC (Fig. 2f, bottom panel). We further plotted the horizontal (*x*) and vertical (*y*) positions of single-tracked pMHCs over time (Fig. 2g, top panel) and analyzed the position changes according to the 3-point moving standard deviation (Fig. 2g, bottom panel). The pMHCs in smFRET and the free pMHCs on the lipid bilayer showed dramatic differences in diffusion at the single-molecule level. These differences have been previously used to differentiate TCR-bound pMHCs from unbound pMHCs for kinetic measurements.^19,20^ Our tracking analyses verified that we detected a FRET signal only if a TCR was bound to a pMHC. In other words, smFRET only measured the conformational dynamics of single TCR–pMHC bonds during the bound state, which are independent of those of unbound molecules and bond association/dissociation. Thus, our conformational measurements by smFRET were independent of TCR–pMHC binding affinity and kinetics.

### The effects of TCR microclusters and CD4 binding

**Fig. 3:**
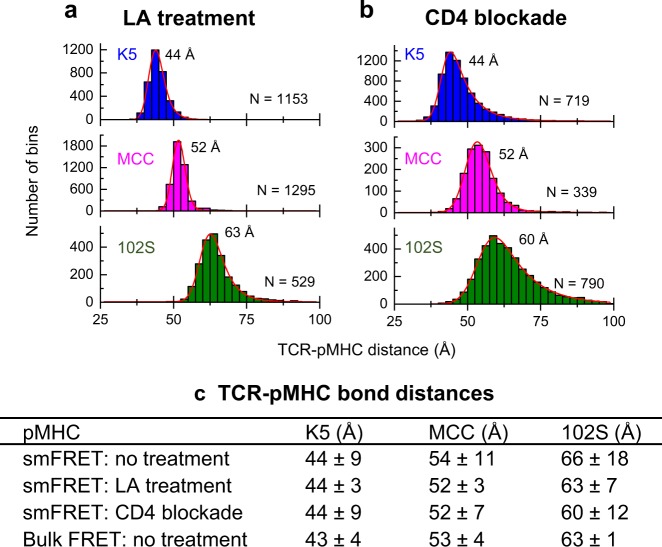
The Effects of TCR microclusters and CD4 binding on TCR–pMHC bond conformation. smFRET TCR–pMHC bond distance measurements in the presence of the actin polymerization inhibitor latrunculin A (LA) (1 µM) (**a**) or 10 µg/mL anti-CD4 blocking antibody (**b**) at 37 °C. In each case, cells were pretreated with LA or antibody for 1 h before introduction to the planar bilayer. Histograms of TCR–pMHC bond distances for the K5, MCC, and 102S pMHCs are shown. Each histogram used 339–1295 single TCR–pMHC bond trajectories to identify the most probable distance for each pMHC. **c** The TCR–pMHC bond distances for each pMHC in different conditions measured by FRET. Data are presented as the most  probable distance ± standard deviation (SD)

TCRs form microclusters upon antigen stimulation (Movie S4). To measure the conformational dynamics of single TCR–pMHC bonds without possible interference from TCR microclusters, we used latrunculin A (LA) to prevent the formation of TCR microclusters during smFRET measurements. Consistent with the results of a previous study, no TCR microclusters were observed at the T-cell synapse after treatment with LA.^21^ We then performed smFRET in the absence of TCR microclusters. We found that the most probale TCR–pMHC bond distances (and distributions) measured in the absence of TCR microclusters were very close to those measured in the presence of TCR microclusters (compare Fig. 2d with Fig. 3), suggesting that our TCR–pMHC bond distance measurements were not affected by TCR microclusters. These results further confirmed that we had determined the true intermolecular TCR–pMHC bond distances at the single-molecule level.

Because the CD4 coreceptor also binds to pMHC, to evaluate the effect of CD4 binding on the TCR–pMHC bond distances, we used an antibody to block CD4 binding to pMHCs and performed smFRET measurements. Our data showed that CD4 binding had little effect on the TCR–pMHC bond distances (compare Fig. 2d and Fig. 3b), which is consistent with the results of previous kinetic studies showing that CD4–pMHC binding is negligible compared to TCR–pMHC interaction in vitro and in situ.^8,22–24^

We then determined the most probable TCR–pMHC bond distances for K5 (super agonist), MCC (agonist), and 102S (weak agonist) under different conditions by smFRET and bulk FRET, as shown in Fig. 3c. We found that neither the disruption of TCR microclusters nor the blocking of CD4 binding changed the TCR–pMHC bond conformation for the three pMHCs. These data (Fig. 3) significantly confirmed the aforementioned smFRET measurements that were made in the absence of any treatments (Fig. 2) and further strengthened our conclusion that the single TCR–pMHC bonds of the three pMHCs have different conformations at the single-molecule level.

### Measurement of intramolecular TCR–CD3ζ conformational changes by FRET

**Fig. 4:**
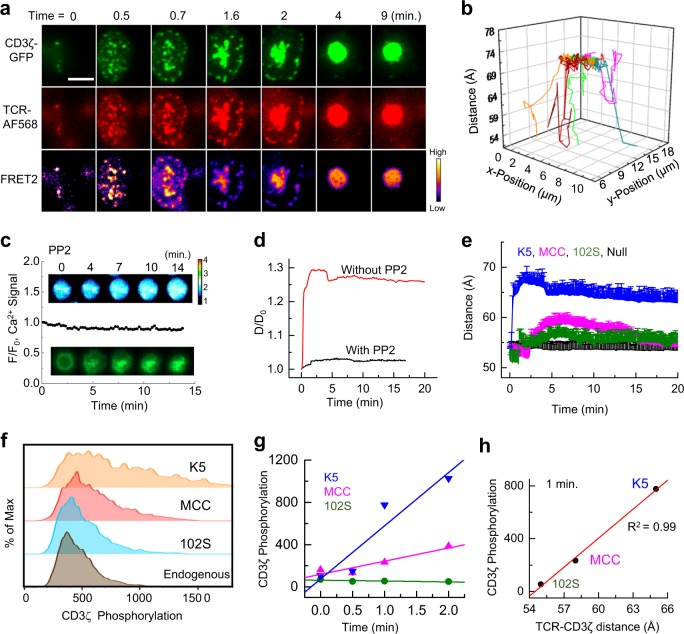
TCR–CD3ζ conformational changes induced by TCR binding. **a** Representative real-time CD3ζ–GFP (green, donor), TCR-Alexa568 (red, acceptor), and FRET (pseudocolor) signals from a typical transmembrane FRET experiment on a lipid bilayer at 37 °C for the K5 pMHC. The same experiments were performed for the MCC and 102S pMHCs. The calculated FRET2 efficiency is indicated in pseudocolor, with the cold-to-hot color spectrum representing weak-to-strong FRET efficiency. Six to eight independent experiments were conducted for each pMHC. Scale bar: 5 µm. **b** 3D illustration of the lateral movement (*x*–*y*) and intramolecular distance change (*z*) between TCR and CD3ζ in the individual TCR/CD3ζ microclusters shown in **a** on the lipid bilayer. The *x* and *y* positions of each microcluster were tracked by the TrackMate plugin in ImageJ, and the *z* positions were calculated according to the corresponding FRET efficiencies. Also see Movie S4 and 5 for microcluster formation, Figs. S9 and S10 for data analysis, Fig. S18 for data calibration and Fig. S12 for the negative controls. **c** Time-lapsed microscopy images of T-cell calcium signaling (top) and CD3ζ clustering (bottom) in the presence of 10 μM PP2. **d** Representative TCR–CD3ζ intramolecular distance changes with (black) and without (red) PP2 treatment. **e** Time-dependent TCR–CD3ζ intramolecular distance changes upon TCR binding to the K5, MCC, 102S, or null pMHC. Each curve shows the average intramolecular distances at consecutive time points measured in three individual microclusters. **f** Phospho-flow cytometry showing the phosphorylation of CD3ζ in T cells upon contact with antigen-presenting cells loaded with K5, MCC, or 102S peptide for 1 min. **g** The time course of CD3ζ phosphorylation upon stimulation with the K5, MCC, or 102S peptide. **h** Correlation between CD3ζ phosphorylation (1 min of stimulation) and TCR–CD3ζ distance

It is not clear what induces the dissociation of CD3ζ from the membrane to initiate T-cell intracellular signaling. To further understand how surface TCR–pMHC bonds propagate extracellular recognition signals to intracellular CD3ζ ITAMs across the cell membrane, we developed a transmembrane FRET assay to measure the conformational change between the extracellular TCR αβ domain and the intracellular CD3ζ chain in the same TCR/CD3ζ complex (Fig. 1a). Upon the addition of 5C.C7 transgenic T cells with Alexa Fluor 568-labeled TCRs and GFP-tagged CD3ζ to a lipid bilayer containing pMHC ligands, we observed rapid microcluster formation and the instant production of transmembrane GFP/Alexa Fluor 568 FRET. The TCRs and CD3ζ were predominantly colocalized in the microclusters [Pearson correlation coefficient,^25^ 0.93 ± 0.07] (Fig. 4a, Figs. S9 and S10, and Movie S5), demonstrating the initiation of T-cell signaling via segregated TCR–pMHC bond-mediated close contacts.^26^ The high level of TCR–CD3ζ colocalization suggested that the obligate assembly of TCR–CD3ζ was necessary for effective T-cell signaling and verified the specificity of transmembrane TCR–CD3ζ FRET. TCRs and CD3ζ microclusters continuously moved from the periphery to the center of the cell and merged into the immunological synapse (Fig. 4a). We tracked and measured the FRET efficiencies of each individual microcluster in real time. After converting the FRET efficiencies to TCR–CD3ζ distances, we plotted a three-dimensional figure (Fig. 4b) to illustrate the simultaneous lateral movement of TCR–CD3ζ complexes (*x*–*y* axis) (also see Fig. S10b) and the intramolecular TCR–CD3ζ distance changes across the cell membrane (*z*-axis) upon K5 pMHC engagement. The intramolecular TCR–CD3ζ distances within different microclusters at equilibrium consistently showed an ~15 Å difference before and after K5 pMHC binding.

To test whether TCR–CD3ζ conformational changes were dependent on TCR signaling, we used an Src kinase inhibitor PP2^27^ to block TCR signaling. Consistent with the results of a previous study,^21^ PP2 completely abrogated T-cell calcium signaling (Fig. 4c, top), while TCRs could still form microclusters (Fig. 4c, bottom). To reveal the role of TCR signaling in TCR–CD3ζ conformational changes, we plotted the time trajectories of the normalized intramolecular TCR–CD3ζ distances against the stimulation time in the absence and presence of PP2 (Fig. 4d). In the absence of PP2, the intracellular CD3ζ chain quickly separated from the TCR extracellular domain upon TCR engagement with the K5 pMHC and then reached a stable, fully extended TCR–CD3ζ conformation. In sharp contrast, the blocking of TCR signaling by PP2 completely abolished the TCR-CD3 conformational change (Fig. 4d). Consistently, this effect of TCR signaling was also found for the MCC and 102S pMHCs (Fig. S11). Together with the results of our negative control experiments (Fig. S12), these PP2 experiments (Fig. 4d and Fig. S11) further verified that transmembrane FRET was specifically caused by the TCR–CD3ζ conformational change.

To compare the conformational dynamics induced by different pMHCs, we plotted the TCR–CD3ζ conformational changes against the stimulation time for the three pMHCs (Fig. 4e). We found that the TCR–CD3ζ conformational change was dependent on the potency of the peptide, as demonstrated by the amplitude and speed of the TCR–CD3ζ conformational change for different peptides (Fig. 4e). Compared with the nonstimulatory null peptide, the super agonist K5 caused the largest change, while the weak agonist 102S caused the smallest conformational change in the TCR–CD3ζ complex. Quantitatively, the K5, MCC, and 102S pMHCs resulted in ~15 Å, ~10 Å, and ~5 Å separations between the TCR and CD3ζ after TCR–pMHC binding, respectively (Fig. 4e). We also found that TCR–CD3ζ conformational changes were restricted in the TCR/CD3ζ microclusters (Fig. 4a and Figs. S3a and S10a). Similar TCR–CD3ζ conformational changes were observed on the glass surface functionalized with pMHCs (Figs. S1a and S13). All together, these data highlighted that the TCR–CD3ζ conformational changes that occurred in the TCR microclusters were driven by TCR–pMHC engagement, which is consistent with previous reports that showed that TCR microclusters are hotspots for TCR signaling.^2,21,28^

We next examined how TCR–CD3ζ conformational changes regulate CD3ζ phosphorylation. Of the three pMHCs tested, we found that the most potent, K5, caused the largest TCR–CD3ζ separation (Fig. 4e) and the highest level of phosphorylation (Fig. 4f, g and Fig. S14). In contrast, the least potent, 102S, caused the smallest TCR–CD3ζ conformational change (Fig. 4e) and resulted in the lowest level of phosphorylation of CD3ζ (Fig. 4f, g and Fig. S14). The CD3ζ conformation and phosphorylation data together suggested that the TCR–CD3ζ conformation controls the degree of the dissociation of CD3ζ from the inner leaflet of the plasma membrane, which influences the temporal (Fig. 4g) and spatial (Fig. 4h) aspects of the phosphorylation of the ITAMs in CD3ζ. Thus, transmembrane FRET could not only directly visualize TCR–CD3ζ conformational changes in situ but could also explain the molecular mechanisms underlying signal propagation in TCR ligand discrimination.

### Linking TCR conformational changes to T-cell responsiveness

**Fig. 5:**
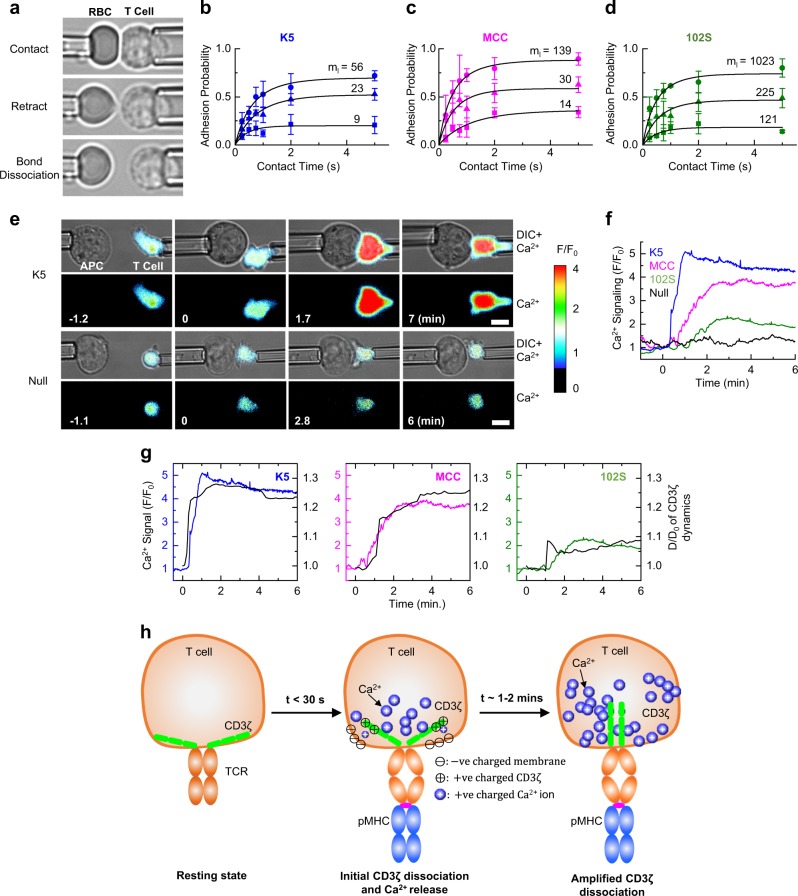
2D kinetics and T-cell Ca^2+^ signaling. **a** 2D micropipette adhesion frequency assay. A micropipette-aspirated T cell (right) was driven by a piezoelectric translator to make controlled contact with an RBC coated with pMHC held by another pipette (left). The retraction of T cells to the starting position resulted in elongation of the RBC, enabling the visual detection of the TCR–pMHC bond. Also see Fig. S15 and Movie S6. Adhesion curves for 5C.C7 TCRs interacting with the K5 (**b**), MCC (**c**), and 102S (**d**) pMHCs measured by micropipette assay at 25 °C at the indicated pMHC site densities. Each cell pair was tested 50 times with a given contact duration to estimate the adhesion probability, and three cell pairs were tested for each contact duration to calculate the mean adhesion probability. The data (points) were fitted by a probabilistic kinetic model (curves) to determine the 2D binding kinetics. The data are summarized in Table 1 and Table S1. **e** Real-time single T-cell calcium signaling measured by fluorescence micropipette. A CH27 cell loaded with the K5 (top row) or null peptide (bottom row, control) was precisely controlled to make contact with a primary 5C.C7 T cell loaded with the Fluo-4 calcium indicator at 37 °C. The fluorescence signal was recorded in real time by time-lapsed microscopy, and the fold increase in Ca^2+^ signaling (*F*/*F*_0_) is shown in pseudocolor. Representative Ca^2+^ imaging experiments for the K5 and null peptides consisting of 6–8 independent experiments for each peptide are shown. Also see Fig. S15c, d for the peptides MCC and 102S. See Movie S7 for more data. **f** Representative time trajectories for Ca^2+^ signaling stimulated by the K5, MCC, 102S, and null peptides. Fluorescence intensity values (*F*) at any given timepoint were divided by the initial fluorescence intensity value at time zero (*F*_0_) to obtain the fold increase in Ca^2+^ signaling after cell contact. **g** The time course of Ca^2+^ signaling (colored curves) and TCR–CD3ζ conformational changes (black curves). **h** TCR–CD3ζ conformational changes were caused by a calcium signaling feedback loop. TCR–pMHC bond formation causes CD3ζ dissociation and initiates Ca^2+^ flux, which in turn promotes CD3ζ dissociation by neutralizing the negative charges of the anionic phospholipids in the T-cell membrane to fully expose the ITAMs on CD3ζ to allow phosphorylation

We then designed a series of experiments to test how the TCR conformation controls TCR binding, signaling, and activation. To test how the TCR–pMHC bond conformation regulates the TCR–pMHC interaction, we performed micropipette adhesion assays to measure the in situ two-dimensional (2D) TCR–pMHC binding kinetics and affinities (Fig. 5a–d, Table 1, Fig. S15, Table S1 and Movie S6).^3^ Our data showed that the binding affinity (driven by the on-rate) is well correlated with the pMHC potency (Table 1). As revealed by the association of a shorter TCR–pMHC bond distance (Fig. 2d) with an increased TCR–pMHC binding affinity (Fig. 5b–d and Table 1), our data suggested that a shorter TCR–pMHC bond promotes the formation of a more stable TCR/pMHC complex, which is consistent with the classic bond length theory in chemistry.^29^

**Table 1:** 2D kinetic parameters

Peptide	Sequence	T-cell activation	*A*_*c*_*K*_*a*_ (µm^4^)	*k*_off_ (s^−1^)	*A*_*c*_*K*_on_ (µm^4^ s^−1^)
K5	ANERADLIAYFKAATKF	Super agonist	3.7 ± 0.6 × 10^−4^	1.0 ± 0.3	3.6 ± 0.5 × 10^−5^
MCC	ANERADLIAYLKQATK	Agonist	1.5 ± 0.7 × 10^−4^	1.3 ± 0.7	2.0 ± 1.1 × 10^−5^
102S	ANERADLIAYLKQASK	Weak agonist	1.6 ± 0.7 × 10^−5^	1.5 ± 0.3	2.4 ± 0.8 × 10^−6^

To understand how the TCR–pMHC bond conformation triggers T-cell signaling, we devised a fluorescence micropipette assay to measure real-time T-cell calcium signaling at the single-cell level. Fast single T-cell calcium flux was observed upon T cell/APC contact (bond formation). Consistently, the calcium signaling amplitude and speed were dependent on the peptide potency (Fig. 5e, f, Fig. S15, and Movie S7). To link the TCR conformational change to TCR signaling, we simultaneously plotted the association of the TCR–CD3ζ conformational change with the Ca^2+^ signal against the stimulation time. We found that the CD3ζ conformational change was synchronized with the Ca^2+^ signal for all three different pMHCs (Fig. 5g). Together with the aforementioned smFRET measurements (Fig. 2), our data revealed that the TCR–pMHC bond conformation (Fig. 2d) governs the amplitude of Ca^2+^ release (Fig. 5e), possibly through bond distance-associated mechanical forces.^30,31^ Ca^2+^ release in turn drives the dissociation of positively charged CD3ζ from the negatively charged phospholipids in the plasma membrane^32^ to expose the CD3ζ ITAM domains for phosphorylation via a Ca^2+^ signaling feedback loop (Fig. 5h).

**Fig. 6:**
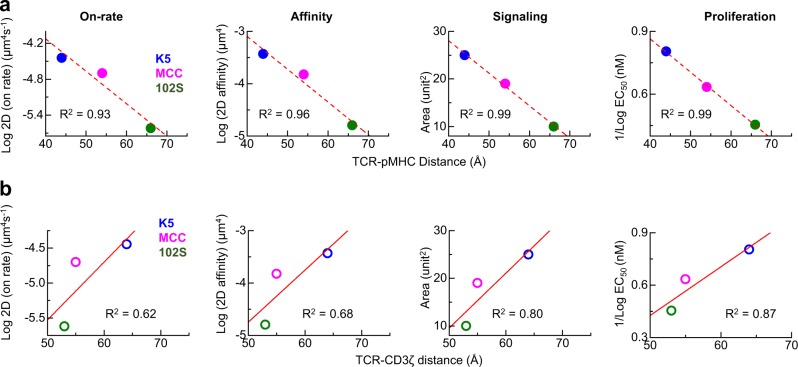
Correlations between TCR conformations and T-cell binding kinetics, signaling, and proliferation. Correlations of the TCR–pMHC bond conformation (**a**) and TCR–CD3ζ distance (**b**) with the 2D on-rate, 2D affinity, calcium signaling, and proliferation for the K5, MCC, and 102S peptides. The EC_50_ data for cell proliferation were adapted from Corse et al.^12^ Data points were fitted with a linear function, and the goodness of correlation is indicated by the *R*^2^ value

According to the values for half-maximal T-cell proliferation (EC_50_) and the 3D half-life of pMHC tetramer binding reported by Corse et al.,^12^ we then plotted the intermolecular TCR–pMHC conformations and the intramolecular TCR–CD3ζ distances against the values for the 2D on-rate, affinity, signaling, and proliferation and the 3D tetramer half-life (Fig. S16). Strong negative (Fig. 6a, solid dots and dashed lines) and positive (Fig. 6b, open dots and solid lines) correlations were found for the TCR–pMHC bond distances and the TCR–CD3ζ distances vs. all of the metrics of TCR binding, signaling, and activation, which govern the entire process of signal reception, transduction, and regulation, respectively. These measurements and correlations indicated the direct physiological relevance of the conformational dynamics of single TCR–pMHC bonds and individual TCR–CD3ζ complexes.

## Discussion

There is considerable controversy about TCR recognition and the initiation of signaling, which are key steps by which a TCR specifically and sensitively recognizes its agonist ligand and then transduces the recognition signal across the plasma membrane to cause alternations in the cytoplasmic portions of the associated CD3 signaling domains. To elucidate the molecular mechanisms underlying this critical process, we need to understand: (1) how the TCR discriminates between signals from a single agonist and those derived from the surrounding abundant self-peptides, and (2) how the TCR precisely propagates such a signal to CD3 to properly trigger T-cell activation. Here, we developed an in situ FRET method to measure the conformational changes of single TCR–pMHC bonds on the cell surface and individual TCR–CD3ζ complexes across the cell membrane (Fig. 1a). Cell surface FRET1 revealed signal generation, and transmembrane FRET2 showed further signal propagation and amplification. The integration of both types of FRET could show the entire process of signal initiation for TCR triggering.

The TCR conformational change model has long been used to explain the mechanism underlying TCR triggering. However, neither crystal structures nor other biochemical assays have been able to provide solid evidence with enough spatiotemporal resolution to either prove or disprove this model. Here, we measured the intermolecular distances of single TCR–pMHC bonds using highly sensitive smFRET (Fig. 1a). In biochemistry, bond length, which is the average distance between the nuclei of two bonded atoms in a molecule, is used to describe the compactness of a bond. Because the TCR was labeled with a scFv and CD4 was also bound to the pMHC, the distance measured here (Fig. 2d) did not reflect the bond length. However, the same scFv (Fig. 1a) was used for all three pMHCs, and CD4 binding contributed negligibly to the TCR–pMHC bond distances (Fig. 3b). The TCR–pMHC bond distances (Fig. 2d) measured in this study provided a reasonable approximation to determine the compactness (or bond length) of a TCR–pMHC bond, thus providing the most basic conformational information at the single-molecule level to advance the understanding of the molecular mechanism underlying TCR recognition in situ.

More importantly, the TCR–pMHC bond conformations measured by our smFRET assay were independent of the binding affinity and kinetics, although they were well correlated (Fig. 6). The smFRET assay is highly distance dependent and is only able to detect FRET signals when a TCR binds to a pMHC on the cell surface (Fig. 1b, d; comparison of K5 with null);^8,33^ in other words, smFRET only measured the conformational dynamics of single TCR–pMHC bonds. The bound state was confirmed by the presence of a nearly immobilized pMHC (diffusion coefficient *D*_bound_ ≈ 0) for each smFRET trajectory, compared with the fast diffusion of free pMHCs (Fig. 2f, g and Fig. S8). Such a dramatic diffusion difference has been previously used to differentiate bound and unbound pMHCs at the single-molecule level.^19,20^ By definition, the affinity is the ratio between bound and unbound molecules at the equilibrium phase, and kinetics describes the rates of bond association/dissociation. Because the TCR–pMHC bond conformation is not influenced by unbound molecules and bond association/dissociation, it is independent of the TCR–pMHC binding affinity and kinetics. In other words, the TCR–pMHC bond conformation measured by smFRET reveals an intrinsic property of the TCR/pMHC complex in the bound state.

Based on the observation that TCRs form microclusters during antigen recognition by conventional TIRF and superresolution microscopy,^21,28,34^ serial engagement has been proposed to explain the high sensitivity of T-cell recognition.^2,3^ To measure the conformational dynamics of single TCR–pMHC bonds without the possible influence of TCR clustering, we used the actin polymerization inhibitor LA to prevent the formation of TCR microclusters. Consistently, the smFRET measurements showed no differences in the TCR–pMHC bond distances measured in the presence or absence of TCR microclusters (Fig. 3c), which reinforced the importance of the single TCR–pMHC bond conformation in the triggering of T-cell signaling and revealed the high sensitivity of TCR recognition.^1,2^

**Fig. 7:**
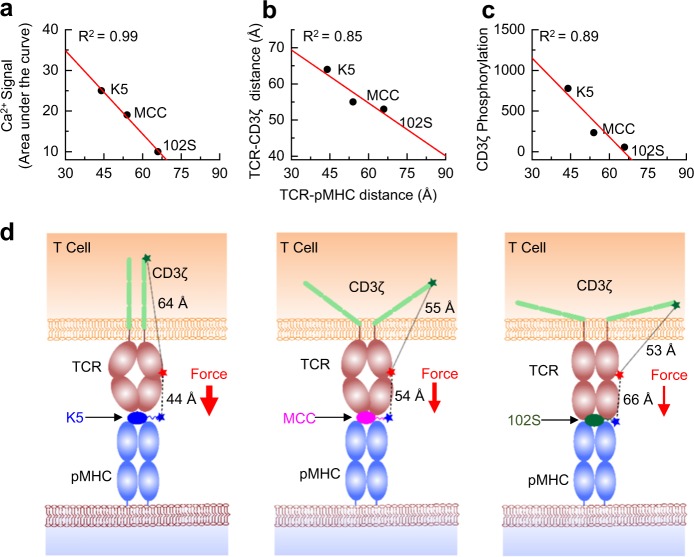
Mechanistic models of TCR triggering and discrimination. Correlations between TCR–pMHC bond conformation and calcium flux (**a**), TCR–CD3ζ distance (**b**), and CD3ζ phosphorylation (**c**). **d** Our proposed model for TCR ligand discrimination. The TCR–pMHC bond conformation controls the TCR–CD3ζ distance to regulate the exposure of ITAMs on CD3ζ for subsequent phosphorylation for the K5, MCC, and 102S pMHCs, respectively

Spatially, our data revealed strong correlations between the intermolecular TCR–pMHC conformation and the Ca^2+^ flux (Fig. 7a), the intramolecular TCR–CD3ζ distance (Fig. 7b), and CD3ζ phosphorylation (Fig. 7c). Our data showed that the strongest agonist, K5, formed the shortest TCR–pMHC bond, which caused the greatest dissociation of CD3ζ from the inner leaflet of the plasma membrane and lead to the maximum exposure of its ITAMs to result in the highest level of phosphorylation. In contrast, a structurally similar but weaker ligand, 102S, formed the longest bond with the TCR, resulting in the least dissociation of CD3ζ from the membrane and the lowest level of phosphorylation. Our results collectively showed that a TCR discriminates between closely related peptides by forming TCR–pMHC bonds with different conformations, which precisely control the accessibility of CD3ζ ITAMs to phosphorylation (Fig. 7d). This discovery highlighted the critical importance of bond conformation in TCR triggering. Physiologically, our study suggested that a short bond, but not a long bond, can efficiently exclude the large, inhibitory tyrosine phosphatase CD45 to increase the phosphorylation of CD3ζ ITAMs by Lck.^5,26^ As bond conformation is independent of binding affinity and kinetics, the results of our study are also very well aligned with those of a previous study showing that elongating the pMHC ectodomain greatly reduces TCR triggering without affecting TCR–pMHC binding.^17^

Temporally, our results revealed that near-instantaneous TCR–pMHC binding (Fig. 2b) was concurrently followed by delayed Ca^2+^ flux and CD3ζ disassociation (Fig. 5g), which was in line with the observation in a previous work that Ca^2+^ and CD3ε/ζ phosphorylation use a positive feedback loop to amplify and sustain T-cell signaling.^32^ Together with the results of previous studies, our data suggested a “TCR–pMHC bond conformational change model” (Fig. 7d) in which a TCR deciphers the structural differences between foreign and self-peptides by forming TCR–pMHC bonds with different distances, which most likely trigger an increase in Ca^2+^ signaling that is proportional to the bond distance through bond distance-controlled mechanical forces;^3,30,31,35^ a previous study has shown that mechanical forces are required to trigger Ca^2+^ flux.^30^ In turn, the released Ca^2+^ regulates the dissociation of positively charged CD3ζ cytoplasmic domains from negatively charged phospholipids in the plasma membrane (Fig. 5h). Such a Ca^2+^ positive signaling feedback loop propagates and amplifies the differences between foreign and self-peptides until TCR–pMHC bond dissociation occurs.^36^ This bond conformational change model (Fig. 7d) is compatible with existing TCR triggering models^5,6^ and explains the high specificity and sensitivity of T-cell recognition and the results of previous studies of CD3 conformational changes.^9,37–40^ Our model suggests that T cells use accurate, reliable, and efficient machinery to faithfully transduce extracellular TCR–pMHC binding into appropriate intracellular signals to ensure the optimal spatial and temporal activation of T cells.

In summary, our study revealed the dynamic process underlying the manner in which TCR recognition signals are initiated, controlled, transmitted, and amplified via the direct linking of the intermolecular TCR–pMHC conformation and the intramolecular TCR–CD3ζ distance to T-cell surface binding, intracellular signaling, and functional responses. This sheds light on the molecular mechanisms by which a TCR deciphers the structural differences between foreign and self-peptides via the TCR–pMHC bond conformation to initiate and amplify different TCR signaling responses for ligand discrimination.

## Experimental procedures

### Mice

The Institutional Animal Care and Use Committee of the University of Chicago approved the animal protocols used in this study. The 5C.C7 TCR transgenic RAG2 knockout mice with a B10.A background were a generous gift from the NIAID.

### Cells

T-cell blasts were obtained by stimulating splenocytes isolated from 5C.C7 TCR transgenic mice with 10 μM MCC peptide (amino acids 88–103, ANERADLIAYLKQATK) according to a protocol approved by the Institutional Animal Care and Use Committee of the University of Chicago. The T-cell blasts were maintained in complete medium (RPMI 1640 medium with 10% fetal bovine serum (FBS), 2 mM l-glutamine, 50 μM β-mercaptoethanol, and penicillin–streptomycin). The T cells were used on days 6–9 for imaging and the micropipette experiments. The live T cells were separated from the dead cells by Ficoll-Paque density gradient media (GE). B-cell lymphoma CH27 cells were used as APCs. The APCs were maintained in the same medium that was used for the T cells.^2^ Human RBCs were isolated from the whole blood of healthy donors from the hospital at the University of Chicago according to a protocol approved by the Institutional Review Board of the University of Chicago.

### Reagents

1-palmitoyl-2-oleoyl-*sn*-glycero-3-phosphocholine (POPC), 1,2-dioleoyl-*sn*-glycero-3-[(N-(5-amino-1-carboxypentyl) iminodiacetic acid) succinyl] nickel salt (DGS-NTA-Ni^2+^), and 1,2-dioleoyl-*sn*-glycero-3-phosphoethanolamine-N [methoxy (polyethyleneglycol)-5000] ammonium salt (PEG5000 PE) were purchased from Avanti Polar Lipids. PBS, BSA, FBS, and Alexa Fluor 568 C5 maleimide were purchased from Thermo Fisher Scientific. PEG-NTA-Ni^2+^-coated coverslips were purchased from MicroSurfaces, Inc. Nunc Lab-Tek eight-well chambered coverslips were purchased from Thermo Fisher Scientific. His-tagged B7 was generated as previously reported.^8^ His-tagged ICAM-1 was purchased from Sino Biological. Cy3 and Cy5 maleimide mono-reactive dyes were purchased from GE Life Sciences. Alexa Fluor 568, Fura-4, and di-methyl sulfoxide were purchased from Thermo Scientific. LB media was obtained from Fisher Scientific.

### pMHCs

For the FRET measurements between TCRs and pMHCs on the cell surface (FRET1), we generated a peptide-exchangeable CLIP-IE^k^ molecule.^41^ The α and β chains of the CLIP-IE^K^ molecule were cloned into pAC vectors with a gp67 signal sequence (BD Biosciences), acidic or basic leucine zipper sequences and a 6-histidine tag at the C-terminus. Primary baculoviruses were prepared for each chain by cotransfecting the construct with linearized baculovirus DNA (BestBac 2.0, Expression Systems) into Sf9 cells using CellFectin reagent (Thermo Fisher Scientific). The cells were washed and incubated at 27 °C for 1 week. The primary viruses were harvested by centrifugation and the collection of the supernatant. Baculoviruses were amplified to generate higher titers by infecting Sf9 cells for another week. Hi5 cells were coinfected with baculoviruses encoding both chains, and the supernatants were harvested after 65 h of infection. The pH of the supernatant was adjusted to pH 6.9 with HEPES-buffered saline (10 mM HEPES pH 7.2, 150 mM NaCl, and 0.02% NaN_3_), 20 mM imidazole, pH 7.2, 5 mM MgCl_2_, and 0.5 mM NiCl_2_. Ni-NTA agarose (Qiagen) was added to the supernatant and stirred overnight at 4 °C. The supernatant was filtered, the Ni-NTA agarose was collected, and CLIP-IE^K^ was eluted using 200 mM imidazole, pH 7.2, in HBS. The protein fractions were analyzed by SDS-PAGE. CLIP-IE^K^ was purified using Superdex 200 size-exclusion column chromatography and Mono-Q anion exchange chromatography (GE healthcare). The purified fractions were used for peptide loading. According to a method described in a previous publication,^8^ peptides with fluorescent maleimide dye (Cy3) at the C-terminus, including K5(C)-ANERADLIAYFKAATKFGGdSdC, MCC(C)-ANERADLIAYLKQATKGGdSdC, T102S(C)-ANERADLIAYLKQASKGGdSdC, and null(C)-ANERAELIAYLTQAAKGGdSdC, were synthesized, labeled with Cy3, purified by HPLC, and verified by mass spectrometry by the Elim Biopharm Company (CA). The peptides of interest were added to the purified CLIP-IE^k^ protein at a 100-fold molar excess for the peptide exchange reaction. Thrombin (1 U/100 μg of IE^k^) was added and incubated at 37 °C for 1 h. The pH of the solution was decreased by adding MES buffer, pH 6.2, at a final concentration of 30 mM, and the IE^K^ was again incubated at 37 °C overnight. The pH of the protein solution was adjusted with 40 mM HEPES, pH 7.2. Extra peptides and denatured proteins were removed by centrifugation (16,000 × *g* for 30 min at 4 °C) and desalting twice with Zeba Spin Desalting Columns (Thermo Fisher).

For the FRET measurements between TCR and CD3ζ across the cell membrane (FRET2) and the 2D micropipette adhesion assays, IE^k^ pMHC monomers generated by the NIH Tetramer Core Facility were used. Biotinylated monomeric IE^k^ was covalently complexed with the ANERADLIAYFKAATKF (K5), ANERADLIAYLKQATK (MCC), ANERADLIAYLKQASK (102S), and PVSKMRMATPLLMQA (human CLIP 87–101) peptides. The IE^k^ monomers were aliquoted and stored at −80 °C until use.

### Production and labeling of the scFvs

Plasmid constructs encoding two mutants of the anti-TCR scFvs J1 and J3 were obtained as a generous gift from Mark M. Davis at Stanford University.^8^ J1 was used for cell surface FRET1, and J3 was used for the transmembrane FRET2 experiments. To generate the J1 and J3 proteins, BL21 bacteria were transfected with cDNA and cultured on a large scale (2 L each) in the presence of isopropyl β-D-1-thiogalactopyranoside. The bacteria were spun down, and the cell pellets were resuspended with bacterial-protein extraction reagent (Thermo Fisher), lysozyme, and DNase, followed by washing with inclusion wash buffer (100 mM NaCl, 50 mM Tris base, and 0.05% (volume) Triton X-100).^42^ The refolding and purification of the recombinant scFvs were performed using a modified method based on previous publications.^43,44^ In brief, the scFvs were unfolded in the presence of 10 mM β-mercaptoethanol for 2 h at 25 °C (in 100 mL of 100 mM Tris-HCl buffer with pH = 8.0, 6 M GuHCl and 200 mM NaCl), so that they could be refolded to obtain proteins with the correct conformations by stepwise dialysis methods without causing protein oxidation. To remove the reducing agent, the denatured recombinant scFvs were dialyzed against 1 L of 100 mM Tris-HCl, pH 8.0, buffer with 6 M GuHCl and 200 mM NaCl for 15 h at 4 °C with gentle stirring. Then, stepwise dialyses were performed in the same Tris-HCl buffer containing decreasing concentrations of GuHCl (4, 2, 1, 0.5, and 0 M) for 15 h for each step at 4 °C with gentle stirring. During the 1 and 0.5 M dialysis steps, 400 mM l-arginine (Sigma-Aldrich) and 375 μM of oxidized glutathione (Sigma-Aldrich) were added. The final dialysis was performed in buffer without GuHCl for 18 h at 4 °C with gentle stirring. The protein was concentrated and stored at 4 °C prior to long-term storage at −20 °C in the presence of 50% glycerol. The purified scFVs were labeled with Cy5-malimide in the presence of 50 µM Tris-(2-carboxyethyl) phosphine hydrochloride for 2 h at room temperature followed by 12 h of gentle mixing at 4 °C. Then, the labeled scFVs were purified by a resin spin column. J1 and J3 were labeled and purified for the FRET experiments. The binding specificity of each of the scFvs for the 5C.C7 TCRs was confirmed by flow cytometry before the imaging experiments.

### CD3ζ–GFP transduction

Primary 5C.C7 T cells were retrovirally transduced with CD3ζ–GFP according to a previously published method.^45^ Ecotropic platinum-E retroviral packaging cells were transiently transfected with the MIG–CD3ζ–GFP vector by calcium phosphate precipitation. The viral supernatant was harvested twice at 48 and 72 h post transfection, filtered by a 0.2 μm cellulose acetate membrane, and used for the subsequent experiments. Splenocytes isolated from 5C.C7 mice were cultured in RPMI supplemented with 10% FBS, 2 mM glutamine, 50 µM 2-mercaptoethanol, 1 mM HEPES, 1 mM sodium pyruvate, 1× glutamine and nonessential amino acids (Thermo Fisher), 100 U/mL penicillin, 100 µg/mL streptomycin, and 50 µg/mL gentamycin and stimulated with precoated 1.5 μg/mL anti-CD3ε Ab (Clone 145-2C11, University of Chicago Monoclonal Antibody Facility) and 0.5 μg/mL anti-CD28 Ab (Clone 37.51, Biolegend) in the presence of 40 U/mL recombinant human IL-2 (Peprotech). After 24 h of cell activation, 2 mL viral supernatant was added to a well of a Retronectin (Clontech)-precoated (12.5 µg/mL in PBS, incubated at 4 °C overnight) six-well plate and centrifuged for 90 min at 3000 × *g*, and then the stimulated cells were transferred to the plate and centrifuged in the viral supernatant supplemented with 4 μg/mL protamine sulfate at 800 × *g* for 90 min. The transduction rate of CD3ζ–GFP was determined by GFP fluorescence at least 16 h after transduction.

### Lipid bilayer preparation

The glass-supported lipid bilayer preparation method was developed based on previous publications.^25,46^ The lipid layer was generated by mixing POPC (90%), DGS-NTA-Ni^2+^ (9.9%), and PEG500PE (0.1%) in chloroform in clean glass vials. The chloroform was dried with 0.22-µm-filtered N_2_, and then vials containing the lipid layer were kept in a vacuum for 2 h to dry them completely. The lipid layer was then resuspended in filtered PBS buffer (pH 7.4; Clontech) at a concentration of 4 µM/mL. To decrease the sizes of the multilamellar lipid vesicles to generate unilamellar vesicles, the cloudy vesicle solution was repeatedly frozen in liquid nitrogen and thawed in a 37 °C water bath 30 times until the solution became clear. The unilamellar vesicle solution was stored at −80 °C for future experiments. Before each experiment, a tube was centrifuged at 33,000 × *g* for 45 min at 4 °C. The supernatant was incubated for 90 min on an eight-well Lab-Tek chamber coverslip that was thoroughly prewashed twice with 5 N NaOH at 50 °C, followed by washing with PBS twice at 37 °C. The lipid vesicles fused onto the glass surface and formed a glass-supported lipid bilayer. The lipid bilayer was washed three times thoroughly with PBS to remove excess lipids. Then, a mixture of His-tagged, fluorescently labeled pMHCs and His-tagged, nonfluorescent ICAM-1, and B7 was added to the lipid bilayer and incubated for 1 h. After 30 min, the unbound proteins were washed with PBS three times. The bound protein was incubated for another 30 min at 37 °C, and the weakly bound proteins were washed away with PBS three additional times. The protein-bound lipid was incubated with 1% BSA for 20 min to minimize the background fluorescence during microscopy experiments. The excess BSA was washed away three times with PBS. The microscopy experiments were performed using imaging buffer (PBS, pH 7.4, 137 mM NaCl, 5 mM KCl, 1 mM CaCl_2_, 2 mM MgCl_2_, 0.7 mM Na_2_HPO_4_, 6 mM D-glucose, and 1% BSA).

The fluidity and integrity of the lipid bilayer were tested by fluorescence recovery after photobleaching experiments with 32 nM Cy3-labeled pMHC reconstituted on a supportive lipid bilayer (Fig. S2a and Movie S2). The photobleaching was performed by using a high power (60 mW) 532-nm CW laser with 5 s of exposure at the center of the imaging area, and the fluorescence recovery of the lipid bilayer was imaged with a 470 ± 10 nm LED light at 10 s intervals. The power and duration of the laser and LED light excitation were controlled by analog modulation. The diffusion coefficient (*D*) was determined by labeling the lipid bilayer with 1 nM of Cy3-labeled pMHCs, which were excited by a stable 532-nm laser (Fig. S2g). The diffusion of the single pMHCs in the lipid bilayer was tracked in real time and characterized (Fig. S2b–f and Movie S1). The experimentally determined diffusion coefficient *D*_*e*_ was verified by a small-scale simulation *D*_*s*,_ which showed high similarity (Fig. S2h–k). The pMHC diffusion constant was determined by the TrackArt program in MATLAB and was consistent with previously reported values.^47^

### Microscopy

All imaging experiments were performed using our custom-built TIRF and epifluorescence microscope setup, which utilized a Nikon-Ti-E inverted microscope attached to an Optosplit III (CAIRN Research) and an Andor iXon 888 EMCCD camera (1024 × 1024 pixel) (Fig. S1b). The individual of 405, 488, 532, and 647-nm (Cobolt) CW laser lines were aligned to an achromatic fiber port (APC type, 400–700 nm, Thorlabs, Inc.) and then passed through an achromatic polarization-maintaining single-mode fiber to a motorized Nikon TIRF illuminator. The lasers were directed by a custom-built quad-band dichroic mirror (Chroma, ZT405-488-532-640rcp) to the sample through a 1.42 NA 100× TIRF objective. A seven-color solid-state LED light source with bandpass filters was also attached with a liquid light guide to the upper filter cube wheel in the Nikon microscope. The fluorescence from the donor and acceptor passed through a quad-band laser clean-up filter (ZET405-488-532-647m) and then passed through the Optosplit III to separate the emission fluorescence of the FRET donor and the acceptor. In the Optosplit cube, we used different filter sets for the FRET1 and FRET2 experiments. For FRET1 (Cy3-Cy5), we used T640lpxr-UF2 (Chroma) as a dichroic filter, ET585/65 for the Cy3 channel (Chroma), and ET655lp for the Cy5 channel (Chroma) for the individual fluorescence signals. For FRET2 (GFP-Alexa568), we used the dichroic filter T560lpxr-UF2 and two bandpass filters: Chroma ET510/20m for GFP and Chroma ET595/50m for Alexa568. For single-molecule imaging, we used hardware sequencing to obtain the highest frame rate (5–10 ms exposure) using high EM gain. Either hardware sequencing (via the camera) or software sequencing (Micromanager) was utilized with analog modulation to synchronize the image acquisition by the EMCCD camera, which triggered each laser and individual LED source. The donor and acceptor signal channels were physically separated by the beam splitter in the Optosplit III, and both signals were imaged simultaneously on the same image frame. The hardware stage control information and images were acquired by Micromanager.^48^

### FRET experiments

For the intermolecular TCR–pMHC FRET1 measurements, T cells were incubated with a saturated concentration (0.027 µg/µL, Fig. S17a) of Cy5-labeled scFv J1 for 30 min at 4 °C. After three washes, the cells were added to a lipid bilayer containing Cy3-labeled pMHCs (0.3 molecules/μm^2^) to perform smFRET using TIRF microscopy in a stream model. To perform the bulk FRET1 experiments, we used a pMHC density of 95 molecules/μm^2^ in the lipid bilayer. For the intramolecular TCR-CD3 FRET2 measurements, T cells transduced with CD3ζ–GFP were labeled with a saturated concentration (0.027 µg/µL, Fig. S17a) of Alexa568-labeled scFv J3 for 30 min at 4 °C. After three washes, the cells were added to a lipid bilayer containing unlabeled pMHCs to perform transmembrane FRET2 using epifluorescence microscopy.

### Density quantification of the TCRs and pMHCs

The average surface TCR density in the 5C.C7 T cells (labeled with a saturated concentration of H57 scFv) in the contact area between a T cell and the lipid bilayer were quantified based on the fluorescence signal of a single Cy5- or Alexa568-labeled H57 scFv. The molecular density of pMHCs in the lipid bilayer was estimated by dividing the total number of pMHC molecules by the total surface area as described previously.^25^

### CD4 blockade and pharmacological treatments

To measure the TCR–pMHC bond distance by smFRET in the absence of CD4 binding, T cells were incubated with 10 μg/mL anti-CD4 antibody (GK 1.5, Biolegend) and 0.027 µg/µL of Cy5-J1 scFV for 30 min at 4 °C. After three washes, the cells were added to a lipid bilayer containing Cy3-pMHCs, and smFRET assays were performed in the continuous presence of 10 μg/mL anti-CD4 antibody at 37 °C using TIRF microscopy. To measure the TCR–pMHC bond distance by smFRET without the formation of TCR microclusters, T cells were incubated with 1 μM LA (Sigma-Aldrich) and 0.027 µg/µL of Cy5-J1 scFv for 1 h at 25 °C. The cells were washed three times, and smFRET assays were performed with a lipid bilayer containing Cy3-labeled pMHCs in the continuous presence of 1 μM LA^3^ at 37 °C using TIRF microscopy. To measure the TCR–CD3ζ conformational dynamics in the absence of TCR signaling, T cells were incubated with 10 μM PP2 (Sigma-Aldrich) and 0.027 µg/µL of Cy5-J1 scFv for 1 h at 25 °C. The cells were washed three times, and smFRET assays were performed with a lipid bilayer containing Cy3-labeled pMHCs in the continuous presence of 10 μM PP2^49^ at 37 °C using epifluorescence microscopy. In parallel, to demonstrate the role of PP2 in blocking TCR signaling, CD3ζ–GFP T cells were loaded with a calcium indicator dye (X-Rhod-5F, Thermo Fisher Scientific), and the real-time calcium flux was measured at 37 °C using epifluorescence microscopy.

### Anisotropy measurements

Anisotropy measurements (Fig. S17b) were performed as previously described.^50^ Briefly, a polarized 532-nm laser was used to excite the samples, and the emission was split by a Wollaston prism (CAIRN Res.) into parallel and perpendicular polarized components that were imaged simultaneously with an EMCCD. The fluorescence anisotropy was determined by the following equation:^51^

1 $$r(t) = I_\parallel - GI_ \bot /I_\parallel + 2GI_ \bot,$$

where $I_\parallel$ and $I_ \bot$are the fluorescence intensities of the parallel ($\parallel$) and perpendicular $( \bot )$ polarized emission components with respect to the vertically polarized excitation. The *G*-factors were calculated according to previously reported methods.^51^

### 2D fluorescent micropipette assays

The micropipette apparatuses were constructed by using a Leica inverted microscope placed on an anti-vibration table (Newport) equipped with manometer systems to apply suction pressure through glass pipettes (Fig. S15a). Two opposing pipettes were attached to two identical piezoelectric micromanipulators (Sensapex) to control the contacts between a T cell and a pMHC-coated RBC or CH27 APC. In the micropipette apparatus, one of the pipettes was also attached to a PI piezo actuator to allow computer-controlled fine movements for the repeated adhesion test cycles. A cell chamber of the desired size was prepared by cutting a coverslip. The temperature of the cell chamber (37 °C) was controlled by an objective heater (Bioptechs). To avoid medium evaporation during heating, the chamber was sealed with mineral oil (Sigma). The real-time images were acquired by an Andor iXon 888 EMCCD camera with a 100× objective and Micromanager software. For real-time calcium imaging, the sample was illuminated by sequentially triggered exposure to 470 ± 20 nm blue light (Spectra X, Lumencor) and white LED light. The triggering of the light channels and the data acquisition were performed with analog modulation using Micromanager.^48^ For the 2D kinetic measurements, only continuous white LED light was used to detect the adhesion between a T cell and a pMHC-coated RBC.

### 2D micropipette kinetic assays

The 2D micropipette adhesion experiments 3 were performed using T-cell blasts^2^ and pMHC-coated RBCs. Monomeric pMHCs were coated onto RBCs by biotin–streptavidin coupling. RBCs isolated from whole blood were biotinylated using different concentrations of biotin-X-NHS according to the manufacturer’s instructions. Ten microliters of RBC solution (~10 × 10^6^) with 2 mg/mL streptavidin solution (10 µl) were mixed for 30 min at 4 °C and then incubated with 20 μg/mL biotinylated pMHC monomer for 30 min at 4 °C. After each step, the RBCs were washed three times.

To determine the surface TCR and pMHC densities, T cells were incubated with 10 μg/mL of the PE-conjugated anti-mouse TCR β chain antibody clone H57-597 (BD) in 200 μL of FACS buffer (RPMI 1640, 5 mM EDTA, 1% BSA, and 0.02% sodium azide) at 4 °C for 30 min, and the pMHC-coated RBCs were stained with the PE-conjugated anti-IE^k^ clone 14.4.4s (BD). The T cells and RBCs were analyzed by a BD LSRFortessa flow cytometer. The fluorescence intensities were compared with those of standard calibration beads (BD Quantibrite PE Beads, BD) to determine the total number of molecules per cell, which were divided by the cell or bead surface area to obtain the site densities. The apparent surface area of a T cell (523 μm^2^) and an RBC (140 μm^2^) were calculated according to the areas of smooth spheres based on the microscopic measurement of their radii.

T cells were incubated at 37 °C with a saturating concentration (10 µg/mL) of purified anti-mouse CD4 (clone GK 1.5, BD) prior to their addition to the chamber. The RBC was moved in and out of contact with the T cell to maintain specific contact times (0.25, 0.5, 0.75, 1, 2, and 5 s) and area by a computer program. The adhesion events were detected by observing RBC elongation upon cell separation. The contact–retraction cycle was repeated 50 times for each given contact time. The specific adhesion probability (*P*_*a*_) for each contact timepoint was calculated by subtracting the nonspecific adhesion frequency ($P_{\mathrm{nonspecific}}$). The following equations were used to analyze the data.

*P*_*a*_ vs. contact time *t* were fitted using a probabilistic model (Eq. 1):^52^

2 $$P_a = 1 - exp\left\{ { - m_rm_lA_cK_a\left[ {1 - {\it{exp}}\left( { - k_rt} \right)} \right]} \right\},$$

where *K*_*a*_ and *k*_*r*_ are the 2D binding affinity and off-rate, *m*_*r*_ and *m*_*l*_ are the respective TCR and pMHC densities that were measured by flow cytometry, and *A*_*c*_ is the contact area. The curve fitting generates two parameters, the effective 2D affinity *A*_*c*_*K*_*a*_ and the 2D off-rate *k*_*r*_. Its product with the off-rate is the effective 2D on-rate:

3 $$A_ck_{\mathrm{on}} = A_cK_a \times k_r$$

4 $$P_a = \frac{{(P_{\mathrm{measured}} - P_{\mathrm{nonspecific}})}}{{1 - P_{\mathrm{nonspecific}}}},$$

where *P*_nonspecifc_ and *P*_measured_ are the nonspecific adhesion fraction and the total measured adhesion, respectively.

### FRET analysis

FRET is a nonradiative process that originates from the dipole–dipole interaction between the electronic states of the donor and acceptor (11, 33). The energy transfer occurs only when the oscillations of the optically induced electronic coherence of the donor are resonant with the electronic energy gap of the acceptor. The efficiency of energy transfer (*E*_FRET_) is sensitive to the distance between the donor and the acceptor, which is typically in the range of 10–100 Å. The energy transfer efficiency (*E*_FRET_) is generally given by:^11,33^

5 $$E_{\mathrm{FRET}} = \frac{1}{{1 + (r/R_0)^6}},$$

where *r* is the distance between donor (*D*) and acceptor (*A*) and *R*_*0*_ is Förster’s distance. FRET is very sensitive to the distance between the acceptor and the donor, which may change due to conformational dynamics or other factors. The *E*_FRET_ between *D* and *A* was calculated by a ratiometric method to reveal the conformational dynamics using the following equation:^11,33^

6 $$E_{\mathrm{FRET}} = \frac{{I_A}}{{I_A + I_D\gamma }},$$

where *I*_*A*_ and *I*_*D*_ are the fluorescence emission intensities of the acceptor and the donor, respectively, and *γ* is the correction factor, which is determined as the ratio of the detection efficiencies of the acceptor and donor channels according to a previously reported method.^33^ Before *E*_FRET_ calculation, each image was background-corrected, and the bleed-through was corrected according to a previously reported method.^33^ The Alexa Fluor 568 intensity was further corrected according to the scFV dissociation kinetics for FRET2 (Fig. S18).

To obtain the distribution of the FRET efficiencies, *E*_FRET_, and the corresponding distances between a FRET donor and a FRET acceptor, we tracked and measured the fluorescence intensities of single donor and acceptor molecules. For each experiment, image registration was first performed using MATLAB for the images of the corresponding frames from the donor channel and the acceptor channel. The donor and acceptor molecules were identified and tracked using TrackMate in ImageJ until the end of each FRET trajectory. The fluorescence intensities of a donor and an acceptor were measured and background-corrected frame-by-frame. Eqns. 5 and 6 were used to calculate *E*_FRET_ and the corresponding distance for both the FRET1 (Cy3/Cy5) and FRET2 (GFP/Alexa568) experiments. By tracking the trajectories of many individual FRET pairs, we obtained the values and distribution of *E*_FRET_ and the corresponding distance using Eqs. 5 and 6.

For the ensemble FRET1 experiments, because the binding affinities were different for the three pMHCs, their TCR occupancies were different. To calculate the TCR–pMHC bond distance in the bound state, the unbound pMHCs were removed from the FRET calculation.^53^ The bound TCRs were directly measured by Cy3/Cy5 FRET because FRET is highly distance specific^8^ (Fig. 1b, comparison of K5 with null). Only the bound pMHCs and the bound TCRs were used to calculate the FRET efficiency and distance using Eqs. 5 and 6 after background and bleed-through correction.

### PMF analysis

One way to quantify the binding strength is to examine the PMF of the fluctuation of the donor–receptor distance *R*. The PMF in this context is given by:^16,54^

7 $$F\left( R \right) = - k_BT \times ln\left( {P\left( R \right)} \right),$$

where *P*(*R*) is the histogram representing the distance that is an average of the steady-state signals collected from 1500 independent TCR–pMHC bond trajectories. The PMF measures the free energy cost of variation for distance *R*. It is minimized at equilibrium. Its curvature governs the size of the fluctuations. A shallower potential curve implies greater fluctuation and weak binding.

### Microcluster tracking analysis

For the FRET2 analysis, we developed a method to track individual CD3ζ (donor) and TCR (acceptor) microclusters in three dimensions (*x*, *y*, and *z*) using the TrackMate plugin in Fiji.^55^ The track for each individual donor and acceptor cluster gave the lateral movement (*x*–*y* axis) as well as the FRET2 efficiency (TCR–CD3ζ distance, *z*-axis) as the microclusters moved toward the center and formed immunological synapses.

### Measurement of CD3ζ phosphorylation

We used phospho-flow cytometry to measure the phosphorylation of CD3ζ at the single-cell level.^56,57^ CH27 cells were preincubated with 10 μM peptide in complete medium for 3 h at 37 °C. CH27 cells with preincubation were used as a negative control. Peptide-loaded CH27 cells were washed three times.^2^ 5C.C7 T cells were rested in serum-free RPMI medium at 37 °C for 3 h to reduce the background phosphorylation level.^25^ A total of 50,000 peptide-loaded CH27 cells and 50,000 rested 5C.C7 T cells were precooled and mixed in a tube on ice. The tube was centrifuged at 300 × *g* for 1 min at 4 °C to initiate cell–cell contact and immediately transferred to a 37 °C water bath to initiate T-cell stimulation. The stimulation was terminated at the indicated time points with 4% PFA fixation. After 10 min of fixation at room temperature, the cells were washed twice with ice-cold PBS containing 2% BSA and then resuspended in 80% methanol and incubated for 30 min at −20 °C. After washing twice with ice-cold PBS, 0.3 μg/mL Alexa Flour-488-labeled anti-pY142-CD3ζ antibody (BD) was added to a final volume of 100 μL of ice-cold PBS and incubated at 4 °C for 45 min. The cells were washed three times with ice-cold PBS containing 2% BSA and analyzed by flow cytometry. The flow cytometry data were further processed with FlowJo software.

### Ca^2+^ imaging

For the Ca^2+^ flux experiments, T cells (~10^6^) were incubated with 5 μM of the fluorescent dye Fluo-4 AM (Thermo Fisher Scientific) for 30 min in complete RPMI 1640 medium. All the Fluo-4 loading and imaging experiments were performed in the presence of 2.5 mM probenecid. The T cells were washed twice with minimal imaging media (MIM; colorless RPMI with 5% FBS and 10 mM HEPES) and then incubated in MIM for 10 min at 37 °C before data collection.^58^ For imaging, a LEITZ DMIRB Leica Microscope equipped with a 100× objective and an iXON Ultra 888 EMCCD camera were used. The calcium flux imaging acquisition was performed with Micromanager software. For the T cell/APC conjugate experiments, CH27 cells (10^6^) were incubated with 4 μM of each peptide for 4 h at 37 °C and then washed with MIM. The T cells (2 μL) and CH27 cells (2 μL) were added to MIM (300 μL) in the cell chamber. The chamber was sealed using mineral oil on both sides to avoid MIM evaporation. The signals from Fluo-4 were collected at intervals of 100 ms for up to 20 min and postprocessed with Fiji software.

## Supplementary information

Supplementary Figures

Single pMHC molecules diffusion on glass supported lipid bilayer

Fluorescence recovery after photobleaching (FRAP)

Synapse formation

TCR microcluster formation on the lipid bilayer

CD3ζ-GFP microcluster formation on lipid bilayer

Measuring in situ kinetics and affinity of TCR-pMHC interactions by 2D micropipette adhesion assay

Single-cell Ca^2+^ Imaging
