# Supplementary material for: TCR–pMHC bond conformation controls TCR ligand discrimination
Source: Cell Mol Immunol. 2019 Sep 17;17(3):203–17. doi: 10.1038/s41423-019-0273-6 (PMC7052167; doi:10.1038/s41423-019-0273-6)
Supplement: Supplementary file 1 — Supplementary Figures [file 41423_2019_273_MOESM1_ESM.pptx]

## Slide 1
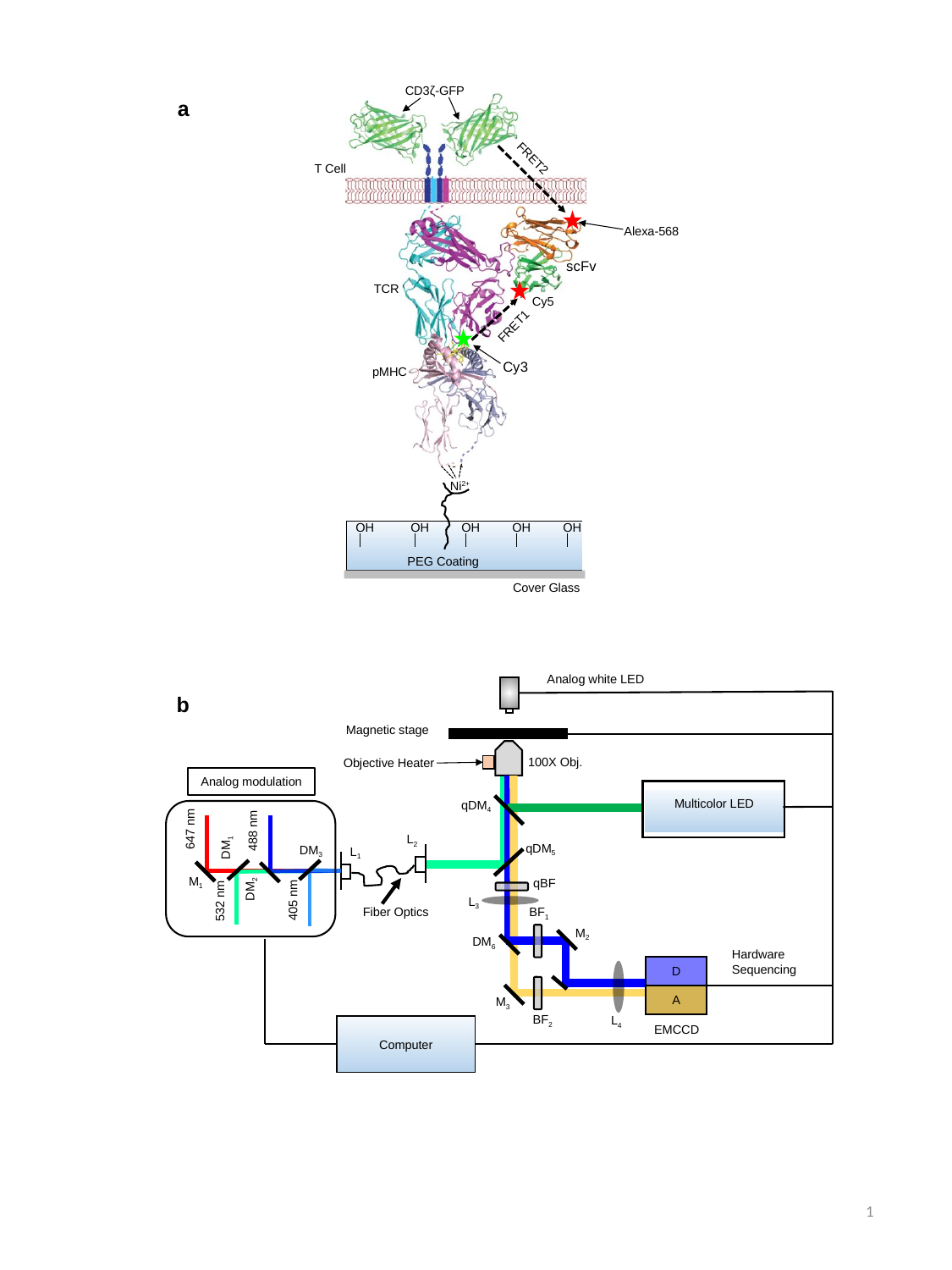

CD3ζ-GFP
FRET2
T Cell
Alexa-568
scFv
TCR
Cy5
FRET1
Cy3
pMHC
Ni2+
OH
OH
OH
OH
OH
PEG Coating
Cover Glass
a
Analog white LED
b
Magnetic stage
100X Obj.
Objective Heater
Analog modulation
Multicolor LED
qDM4
647 nm
488 nm
L2
qDM5
DM1
DM3
L1
M1
qBF
DM2
405 nm
532 nm
L3
Fiber Optics
BF1
M2
DM6
Hardware
Sequencing
D
A
M3
BF2
L4
EMCCD
Computer
1

## Slide 2
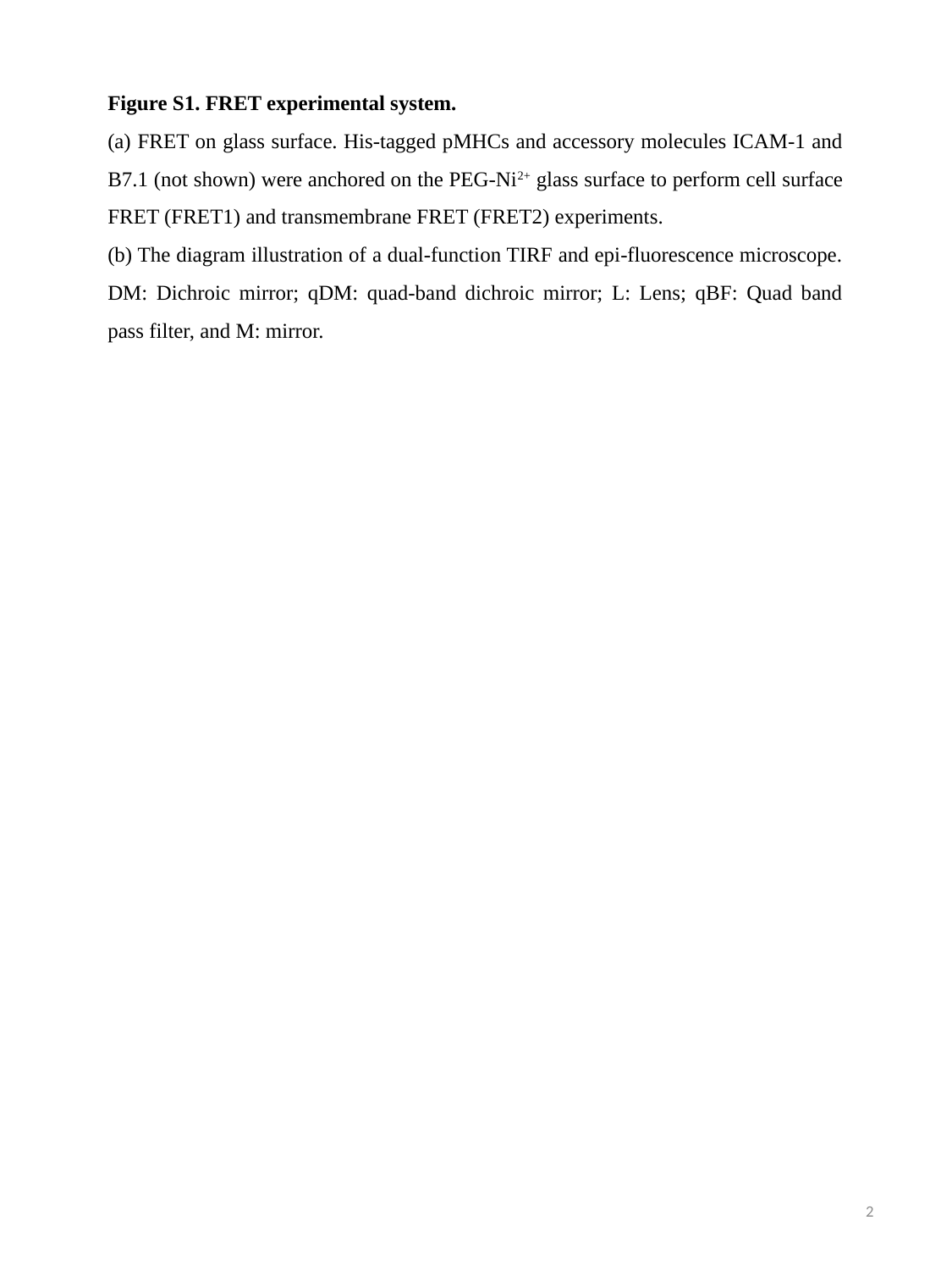

Figure S1. FRET experimental system.
(a) FRET on glass surface. His-tagged pMHCs and accessory molecules ICAM-1 and B7.1 (not shown) were anchored on the PEG-Ni2+ glass surface to perform cell surface FRET (FRET1) and transmembrane FRET (FRET2) experiments.
(b) The diagram illustration of a dual-function TIRF and epi-fluorescence microscope. DM: Dichroic mirror; qDM: quad-band dichroic mirror; L: Lens; qBF: Quad band pass filter, and M: mirror.
2

## Slide 3
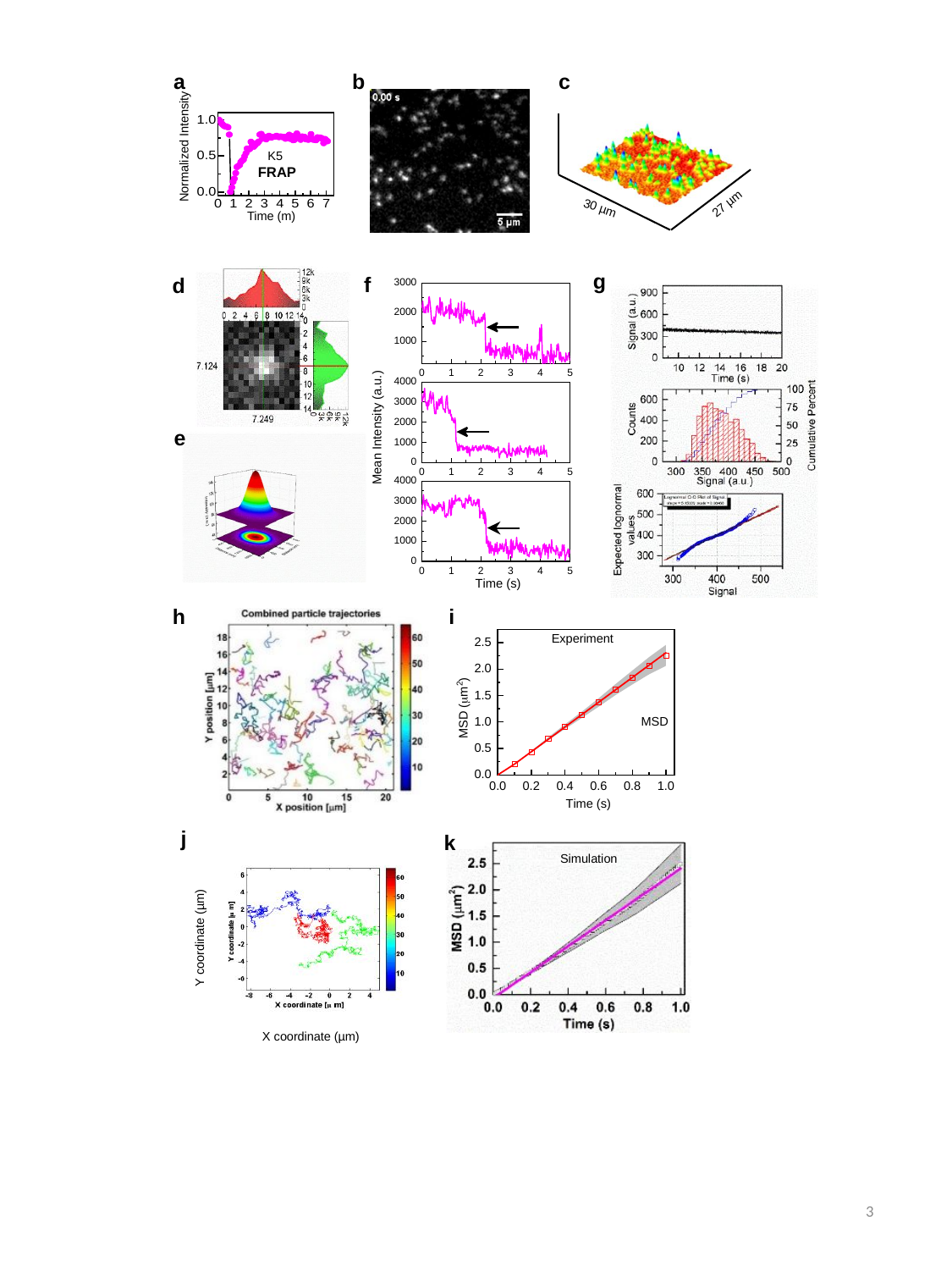

a
b
c
Normalized Intensity
Time (m)
K5
27 µm
30 µm
FRAP
g
f
d
e
h
i
Experiment
MSD
j
k
Y coordinate (µm)
X coordinate (µm)
Simulation
3

## Slide 4
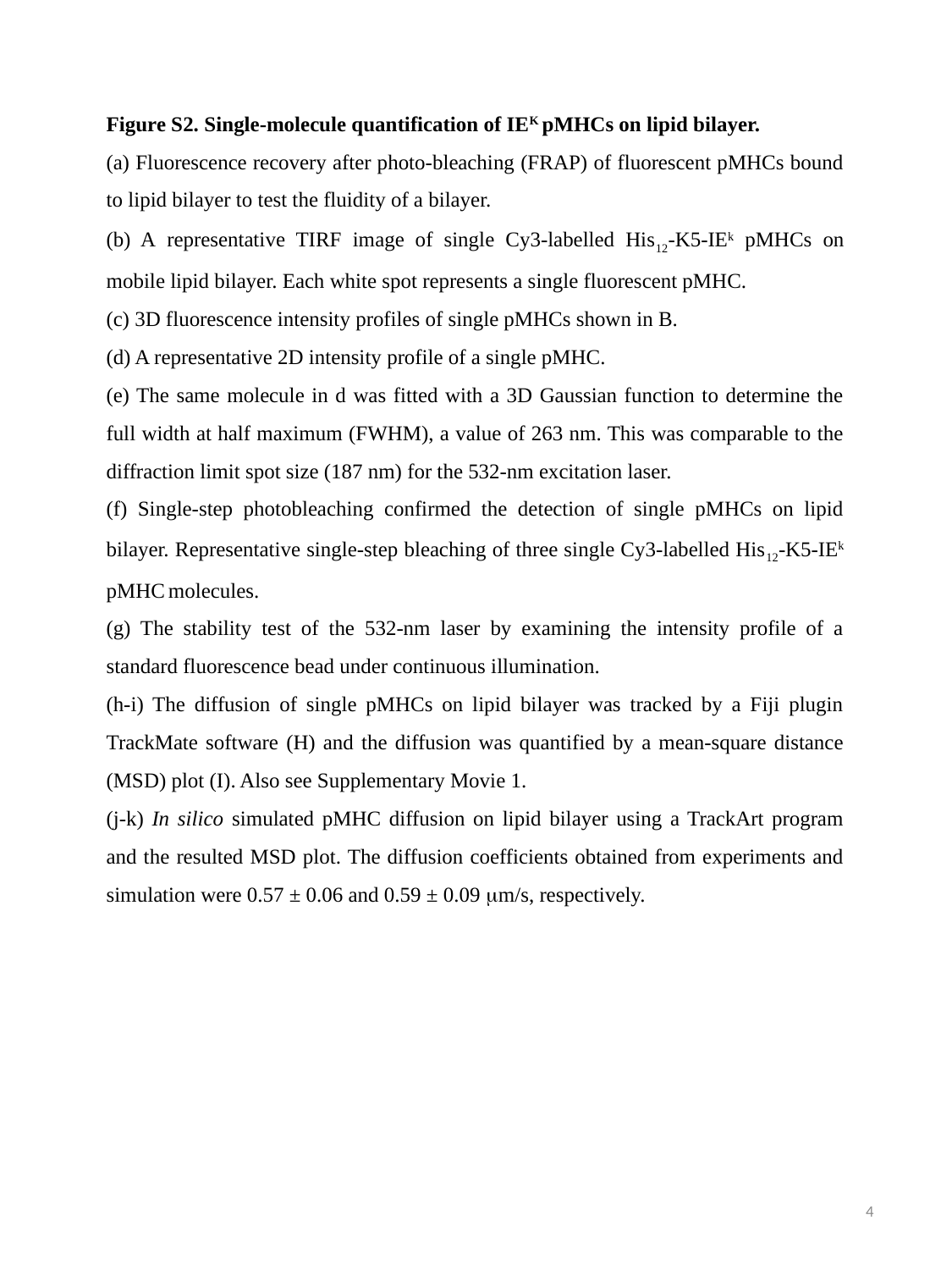

Figure S2. Single-molecule quantification of IEK pMHCs on lipid bilayer.
(a) Fluorescence recovery after photo-bleaching (FRAP) of fluorescent pMHCs bound to lipid bilayer to test the fluidity of a bilayer.
(b) A representative TIRF image of single Cy3-labelled His12-K5-IEk pMHCs on mobile lipid bilayer. Each white spot represents a single fluorescent pMHC.
(c) 3D fluorescence intensity profiles of single pMHCs shown in B.
(d) A representative 2D intensity profile of a single pMHC.
(e) The same molecule in d was fitted with a 3D Gaussian function to determine the full width at half maximum (FWHM), a value of 263 nm. This was comparable to the diffraction limit spot size (187 nm) for the 532-nm excitation laser.
(f) Single-step photobleaching confirmed the detection of single pMHCs on lipid bilayer. Representative single-step bleaching of three single Cy3-labelled His12-K5-IEk pMHC molecules.
(g) The stability test of the 532-nm laser by examining the intensity profile of a standard fluorescence bead under continuous illumination.
(h-i) The diffusion of single pMHCs on lipid bilayer was tracked by a Fiji plugin TrackMate software (H) and the diffusion was quantified by a mean-square distance (MSD) plot (I). Also see Supplementary Movie 1.
(j-k) In silico simulated pMHC diffusion on lipid bilayer using a TrackArt program and the resulted MSD plot. The diffusion coefficients obtained from experiments and simulation were 0.57 ± 0.06 and 0.59 ± 0.09 μm/s, respectively.
4

## Slide 5
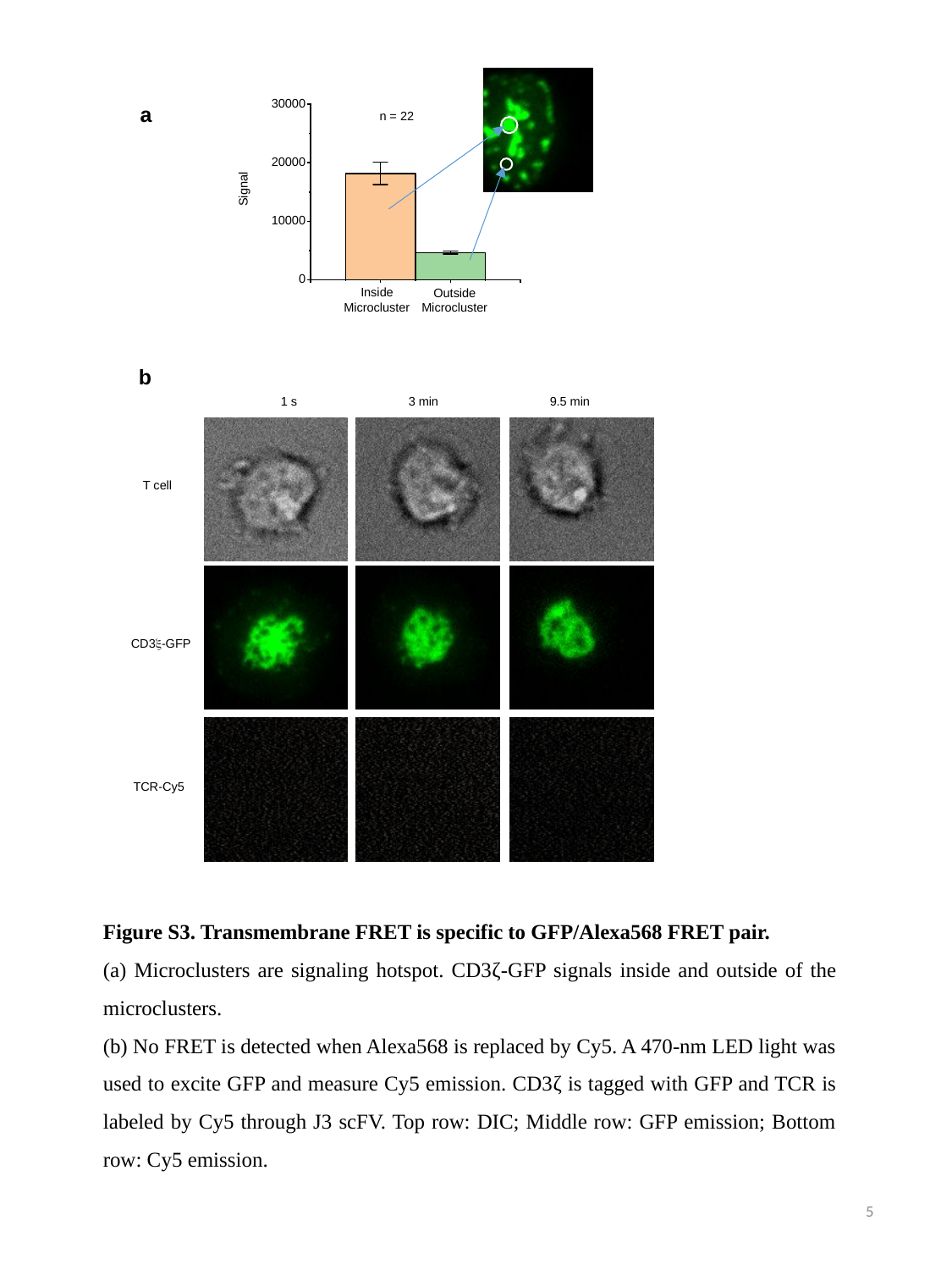

n = 22
Signal
Inside
Microcluster
Outside
Microcluster
a
b
1 s
3 min
9.5 min
T cell
CD3-GFP
TCR-Cy5
Figure S3. Transmembrane FRET is specific to GFP/Alexa568 FRET pair.
(a) Microclusters are signaling hotspot. CD3ζ-GFP signals inside and outside of the microclusters.
(b) No FRET is detected when Alexa568 is replaced by Cy5. A 470-nm LED light was used to excite GFP and measure Cy5 emission. CD3ζ is tagged with GFP and TCR is labeled by Cy5 through J3 scFV. Top row: DIC; Middle row: GFP emission; Bottom row: Cy5 emission.
5

## Slide 6
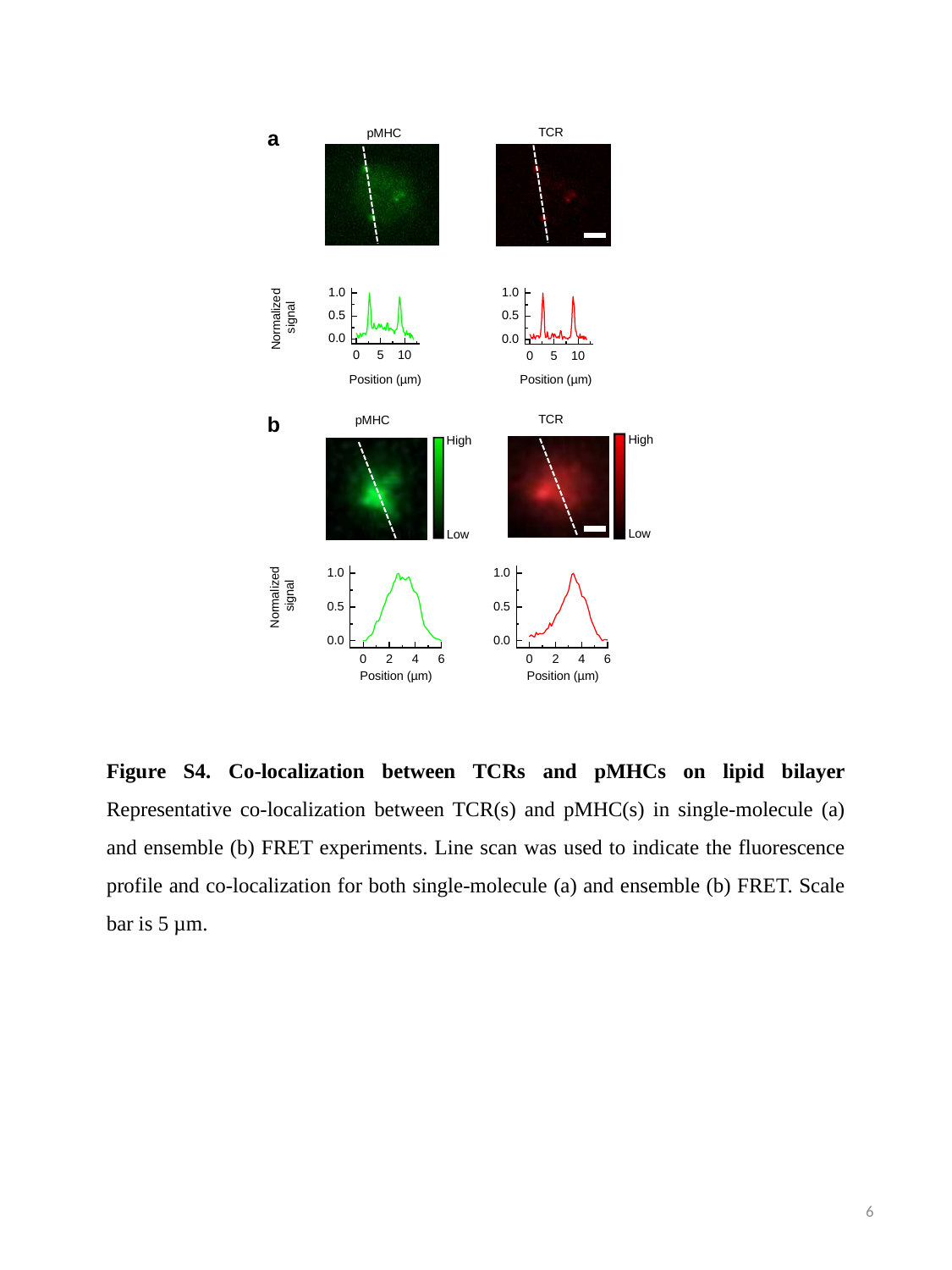

a
TCR
pMHC
Normalized
signal
Position (µm)
b
TCR
pMHC
High
Low
High
Low
Normalized
signal
Position (µm)
Position (µm)
Position (µm)
Figure S4. Co-localization between TCRs and pMHCs on lipid bilayer Representative co-localization between TCR(s) and pMHC(s) in single-molecule (a) and ensemble (b) FRET experiments. Line scan was used to indicate the fluorescence profile and co-localization for both single-molecule (a) and ensemble (b) FRET. Scale bar is 5 µm.
6

## Slide 7
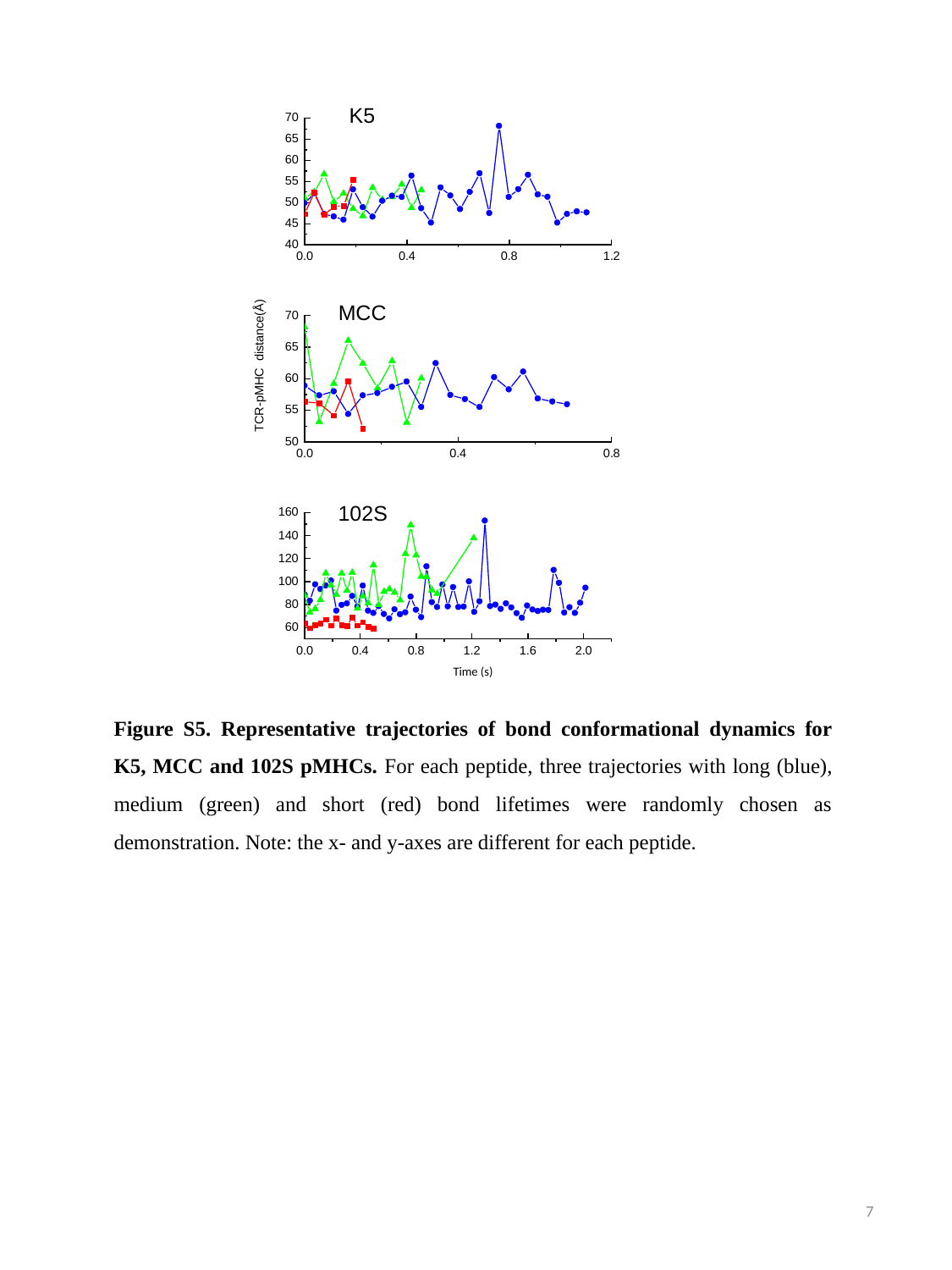

K5
TCR-pMHC distance(Å)
MCC
102S
Time (s)
Figure S5. Representative trajectories of bond conformational dynamics for K5, MCC and 102S pMHCs. For each peptide, three trajectories with long (blue), medium (green) and short (red) bond lifetimes were randomly chosen as demonstration. Note: the x- and y-axes are different for each peptide.
7

## Slide 8
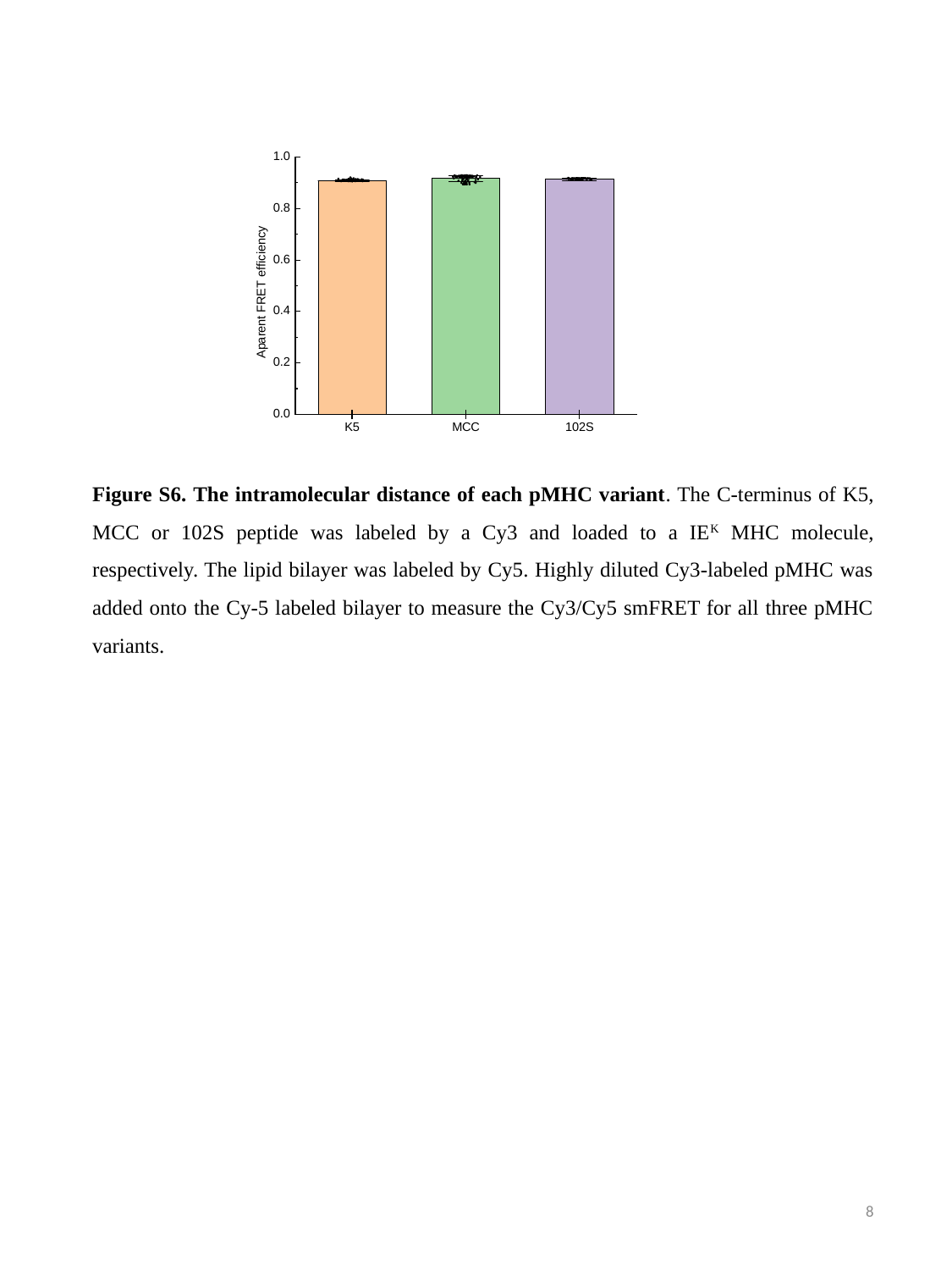

Figure S6. The intramolecular distance of each pMHC variant. The C-terminus of K5, MCC or 102S peptide was labeled by a Cy3 and loaded to a IEK MHC molecule, respectively. The lipid bilayer was labeled by Cy5. Highly diluted Cy3-labeled pMHC was added onto the Cy-5 labeled bilayer to measure the Cy3/Cy5 smFRET for all three pMHC variants.
8

## Slide 9
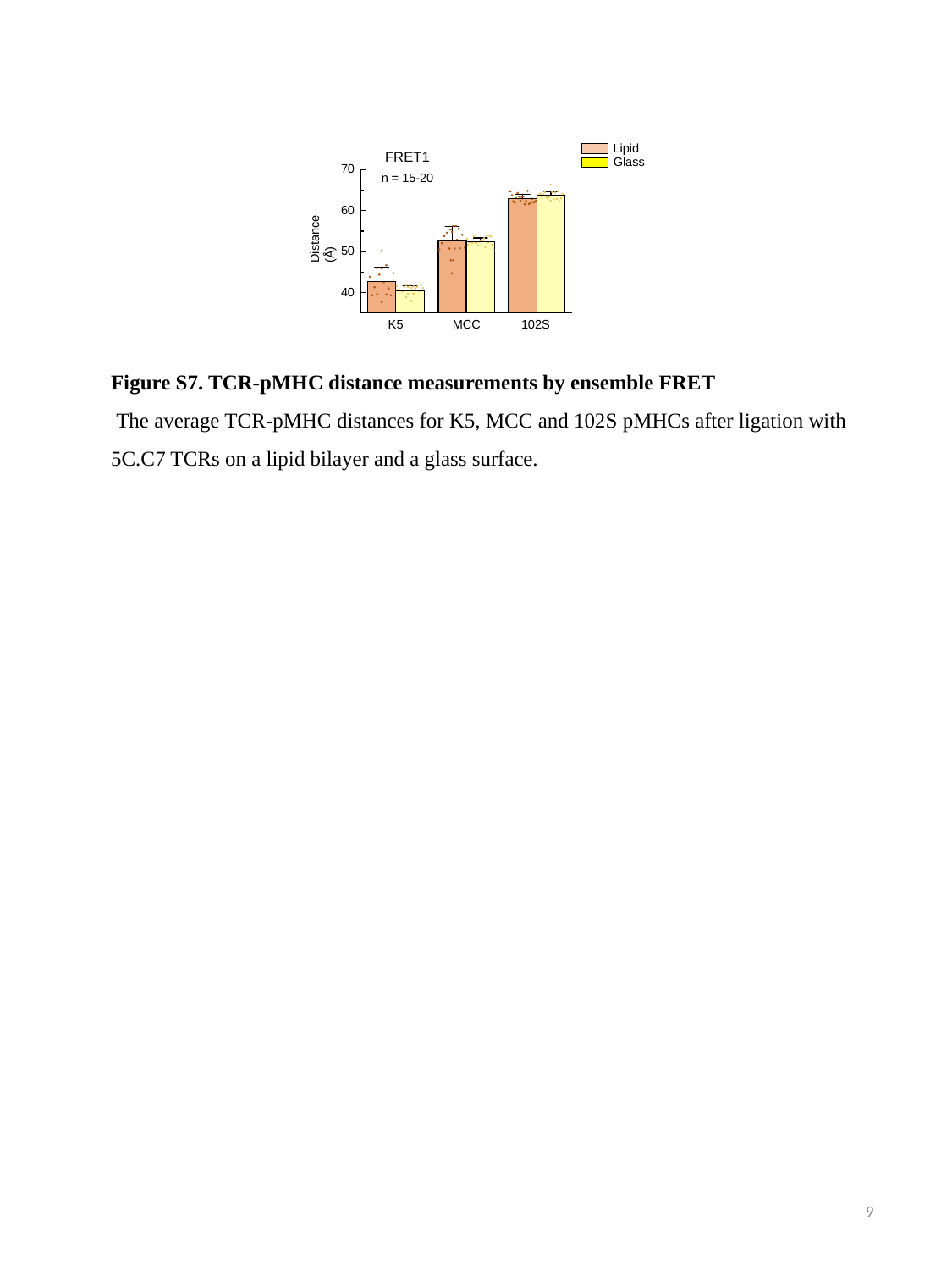

Lipid
Glass
n = 15-20
FRET1
Distance (Å)
K5
MCC
102S
Figure S7. TCR-pMHC distance measurements by ensemble FRET
 The average TCR-pMHC distances for K5, MCC and 102S pMHCs after ligation with 5C.C7 TCRs on a lipid bilayer and a glass surface.
9

## Slide 10
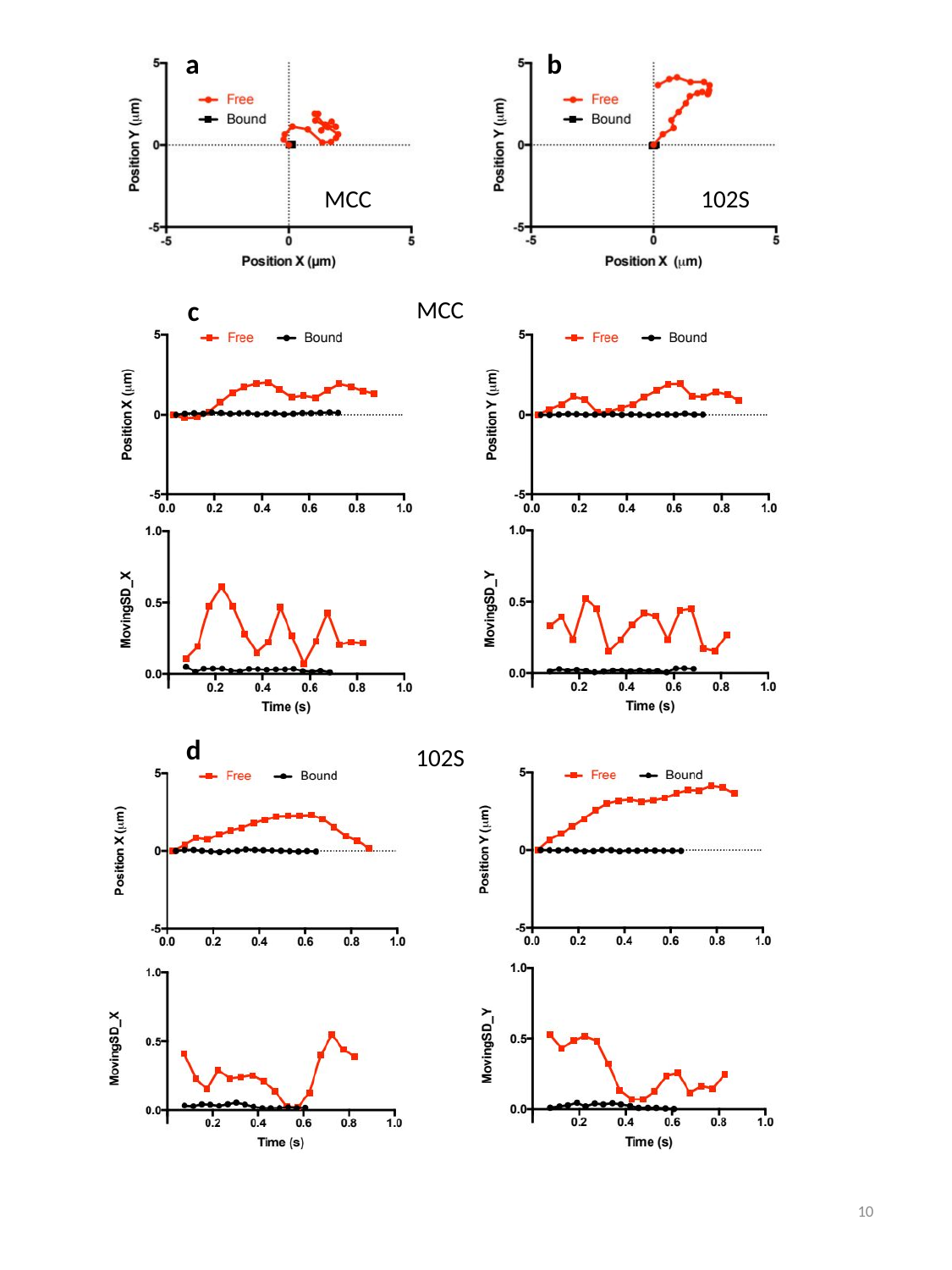

a
b
MCC 102S
c
MCC
d
102S
10

## Slide 11
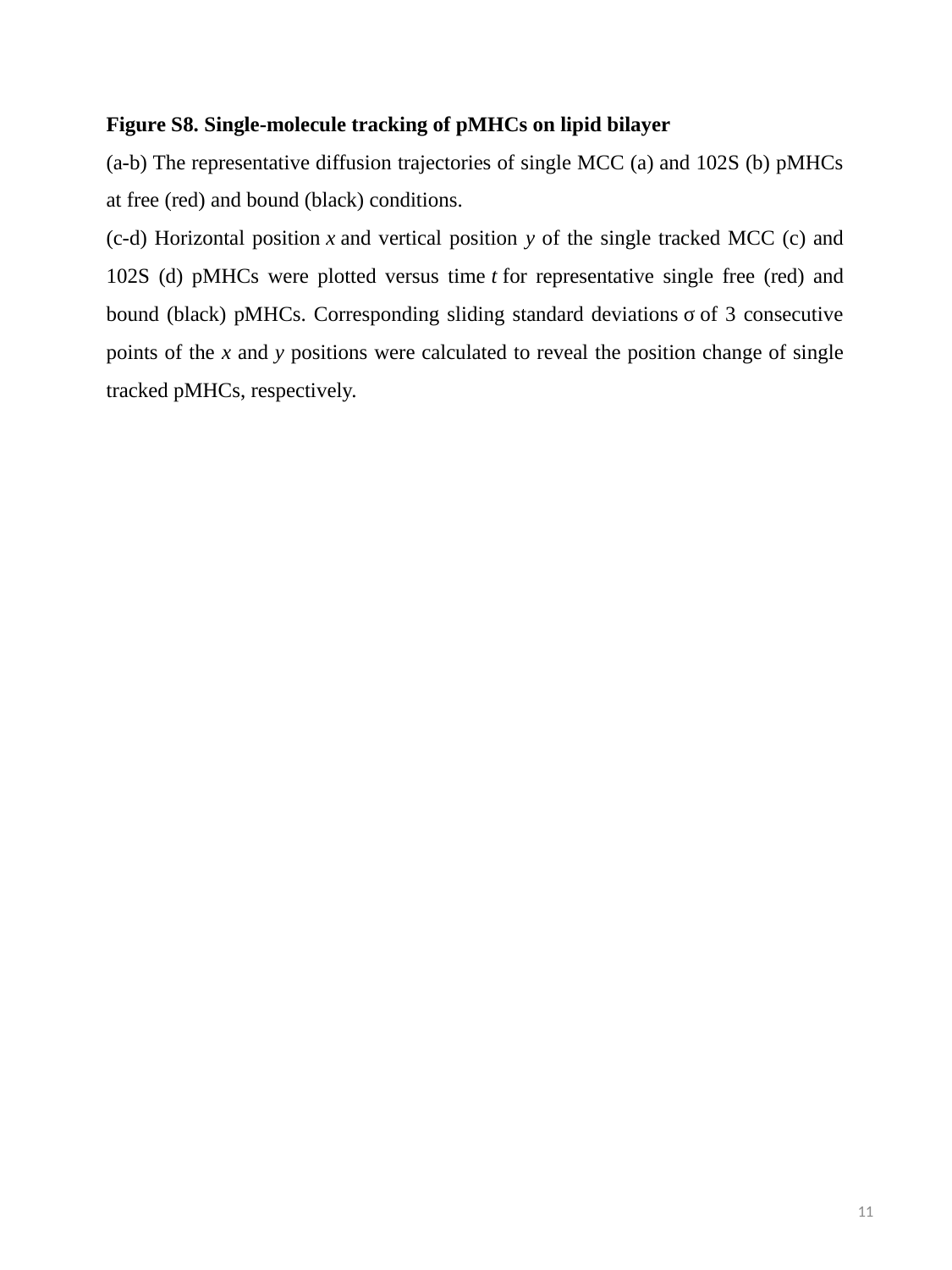

Figure S8. Single-molecule tracking of pMHCs on lipid bilayer
(a-b) The representative diffusion trajectories of single MCC (a) and 102S (b) pMHCs at free (red) and bound (black) conditions.
(c-d) Horizontal position x and vertical position y of the single tracked MCC (c) and 102S (d) pMHCs were plotted versus time t for representative single free (red) and bound (black) pMHCs. Corresponding sliding standard deviations σ of 3 consecutive points of the x and y positions were calculated to reveal the position change of single tracked pMHCs, respectively.
11

## Slide 12
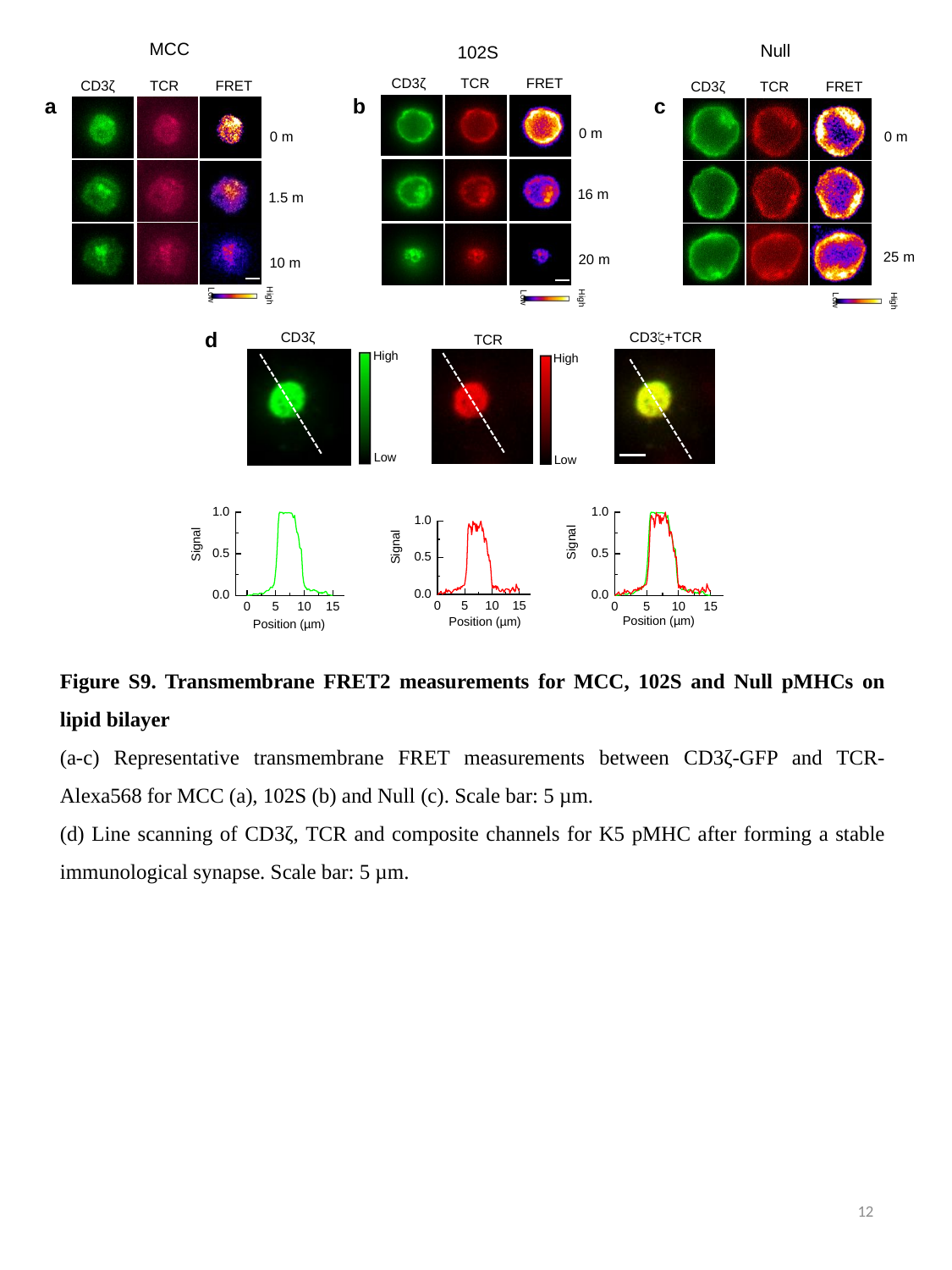

MCC
Null
102S
CD3ζ
TCR
FRET
CD3ζ
TCR
FRET
CD3ζ
TCR
FRET
a
b
c
0 m
16 m
20 m
0 m
25 m
0 m
1.5 m
10 m
High
Low
High
Low
High
Low
d
CD3ζ
CD3+TCR
TCR
High
Low
High
Low
Signal
Signal
Signal
Position (µm)
Position (µm)
Position (µm)
Figure S9. Transmembrane FRET2 measurements for MCC, 102S and Null pMHCs on lipid bilayer
(a-c) Representative transmembrane FRET measurements between CD3ζ-GFP and TCR-Alexa568 for MCC (a), 102S (b) and Null (c). Scale bar: 5 µm.
(d) Line scanning of CD3ζ, TCR and composite channels for K5 pMHC after forming a stable immunological synapse. Scale bar: 5 µm.
12

## Slide 13
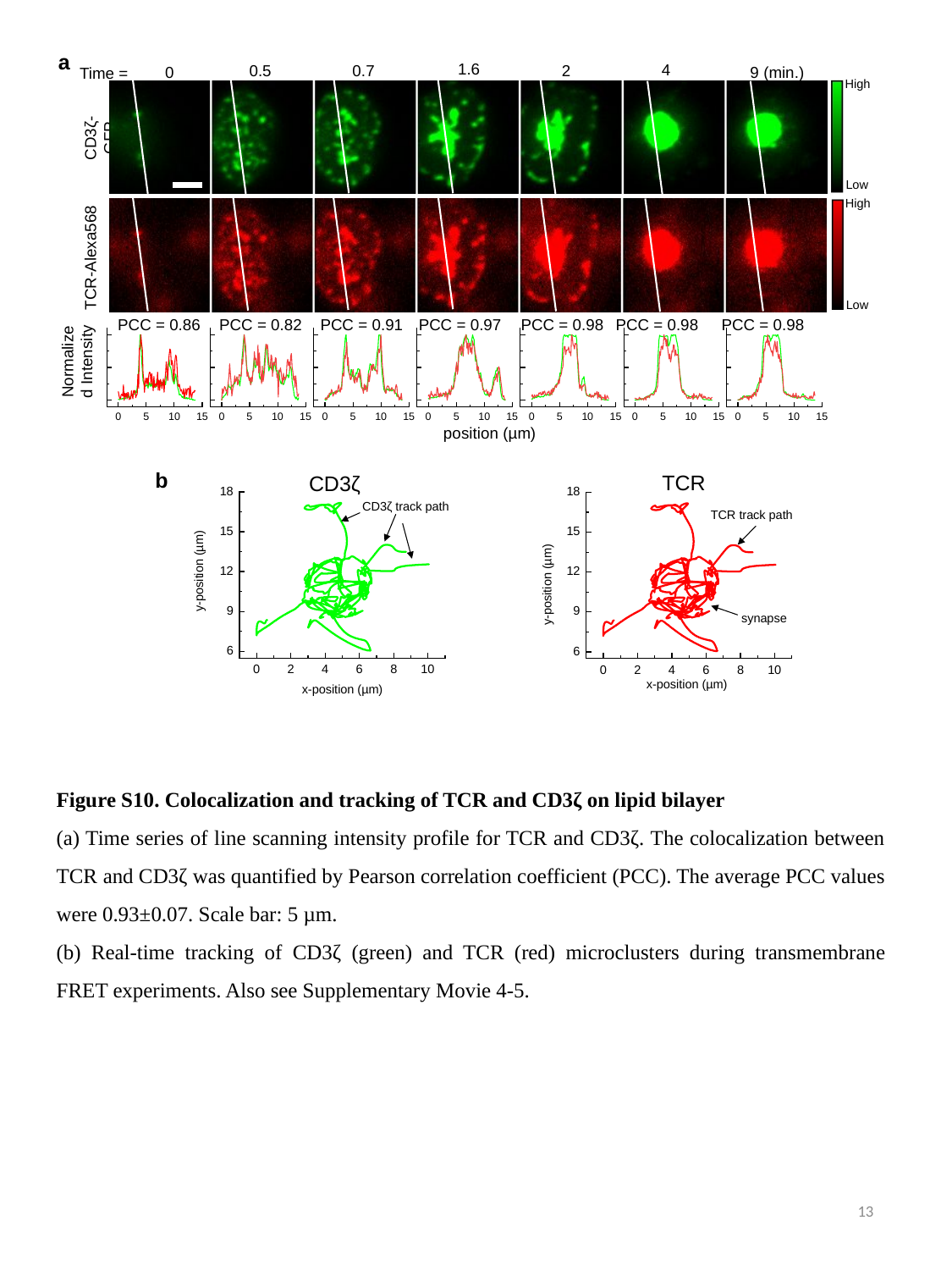

a
1.6
4
0.7
2
0.5
0
Time =
9 (min.)
High
Low
CD3ζ-GFP
TCR-Alexa568
High
Low
PCC = 0.86
PCC = 0.82
PCC = 0.91
PCC = 0.97
PCC = 0.98
PCC = 0.98
PCC = 0.98
Normalized Intensity
position (µm)
b
TCR
TCR track path
y-position (µm)
synapse
CD3ζ
CD3ζ track path
y-position (µm)
x-position (µm)
x-position (µm)
Figure S10. Colocalization and tracking of TCR and CD3ζ on lipid bilayer
(a) Time series of line scanning intensity profile for TCR and CD3ζ. The colocalization between TCR and CD3ζ was quantified by Pearson correlation coefficient (PCC). The average PCC values were 0.93±0.07. Scale bar: 5 µm.
(b) Real-time tracking of CD3ζ (green) and TCR (red) microclusters during transmembrane FRET experiments. Also see Supplementary Movie 4-5.
13

## Slide 14
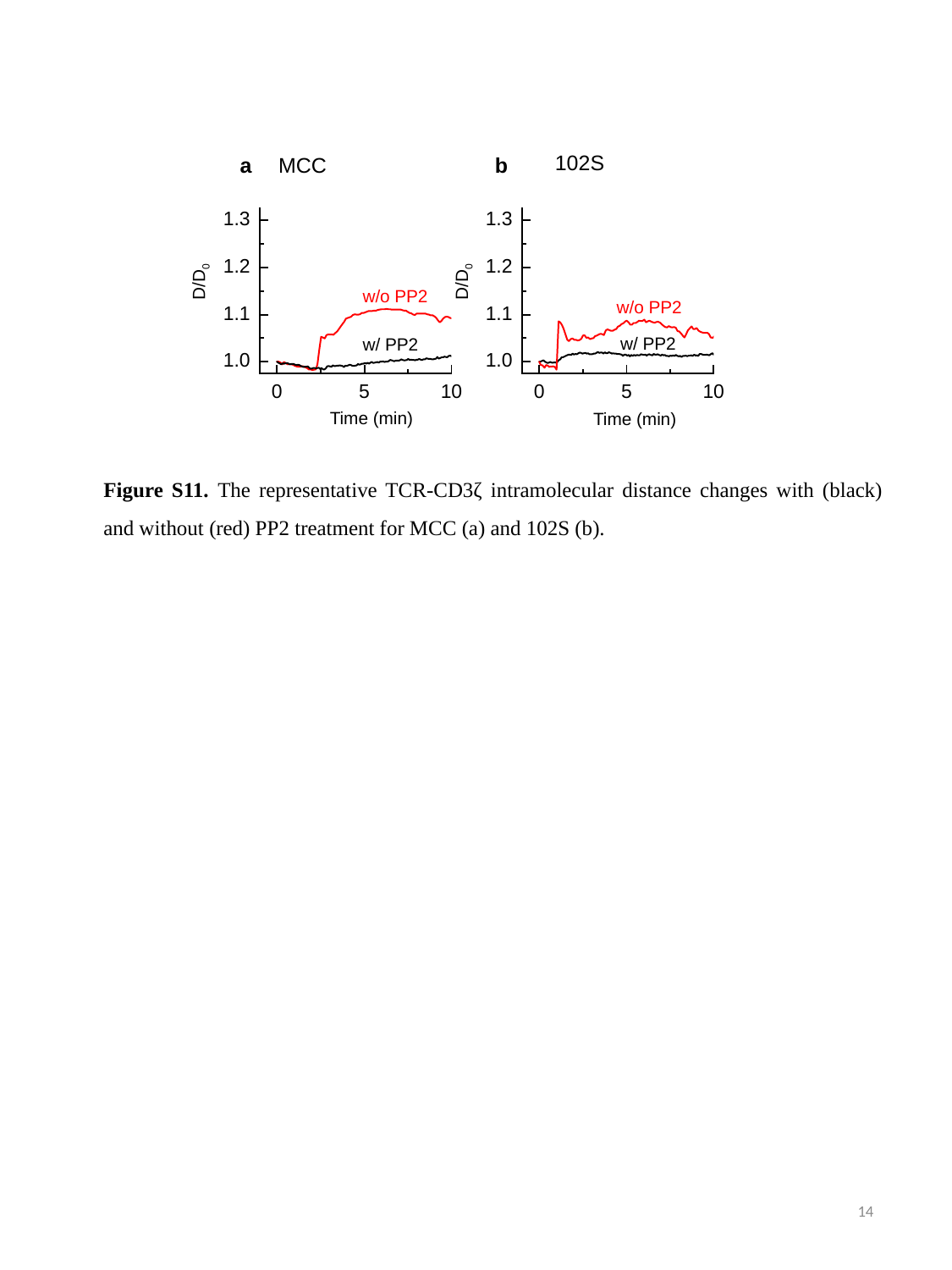

102S
b
a
MCC
D/D0
w/o PP2
w/ PP2
Time (min)
Time (min)
D/D0
w/o PP2
w/ PP2
Figure S11. The representative TCR-CD3ζ intramolecular distance changes with (black) and without (red) PP2 treatment for MCC (a) and 102S (b).
14

## Slide 15
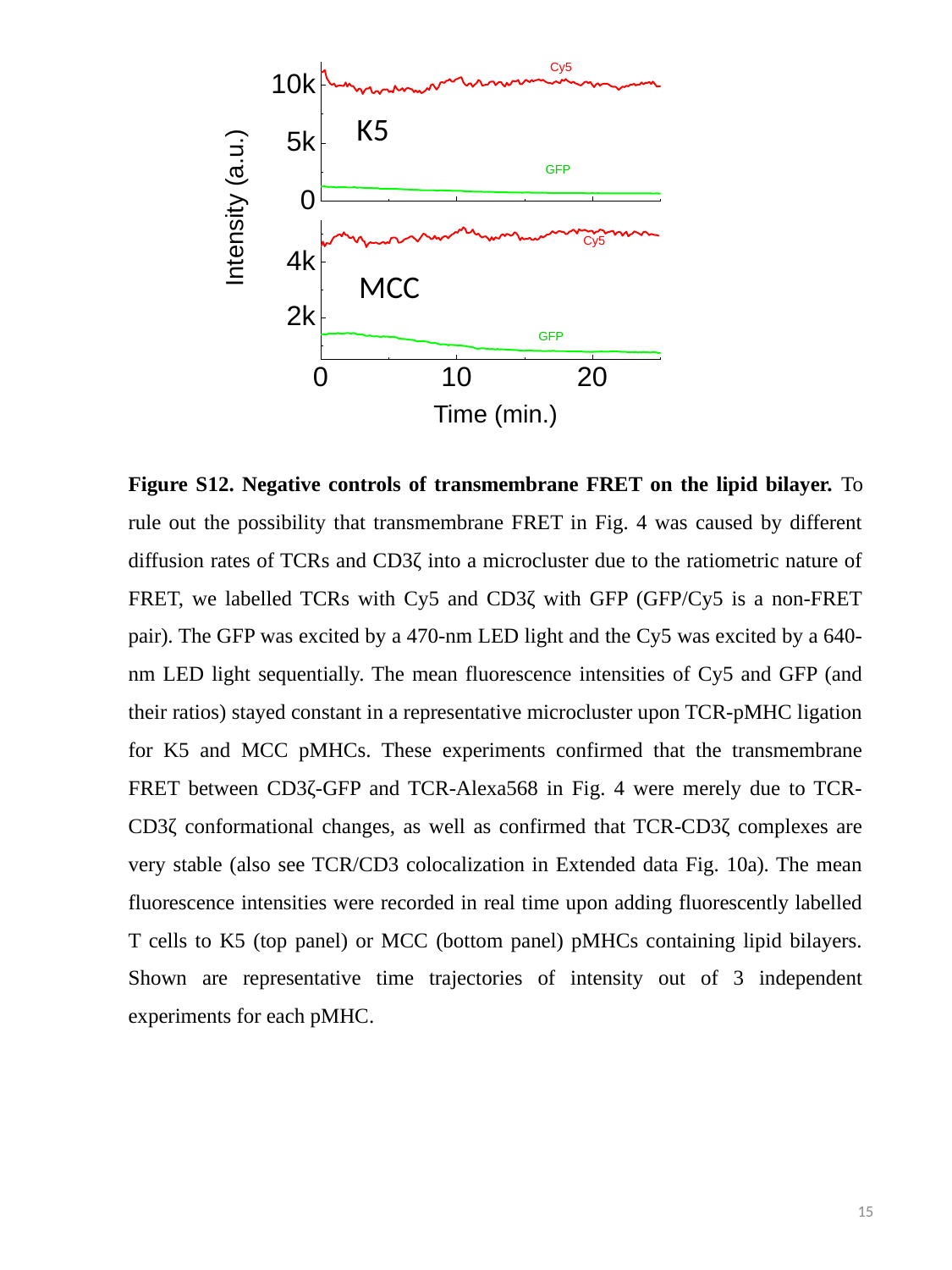

Cy5
Intensity (a.u.)
GFP
Cy5
GFP
Time (min.)
K5
MCC
Figure S12. Negative controls of transmembrane FRET on the lipid bilayer. To rule out the possibility that transmembrane FRET in Fig. 4 was caused by different diffusion rates of TCRs and CD3ζ into a microcluster due to the ratiometric nature of FRET, we labelled TCRs with Cy5 and CD3ζ with GFP (GFP/Cy5 is a non-FRET pair). The GFP was excited by a 470-nm LED light and the Cy5 was excited by a 640-nm LED light sequentially. The mean fluorescence intensities of Cy5 and GFP (and their ratios) stayed constant in a representative microcluster upon TCR-pMHC ligation for K5 and MCC pMHCs. These experiments confirmed that the transmembrane FRET between CD3ζ-GFP and TCR-Alexa568 in Fig. 4 were merely due to TCR-CD3ζ conformational changes, as well as confirmed that TCR-CD3ζ complexes are very stable (also see TCR/CD3 colocalization in Extended data Fig. 10a). The mean fluorescence intensities were recorded in real time upon adding fluorescently labelled T cells to K5 (top panel) or MCC (bottom panel) pMHCs containing lipid bilayers. Shown are representative time trajectories of intensity out of 3 independent experiments for each pMHC.
15

## Slide 16
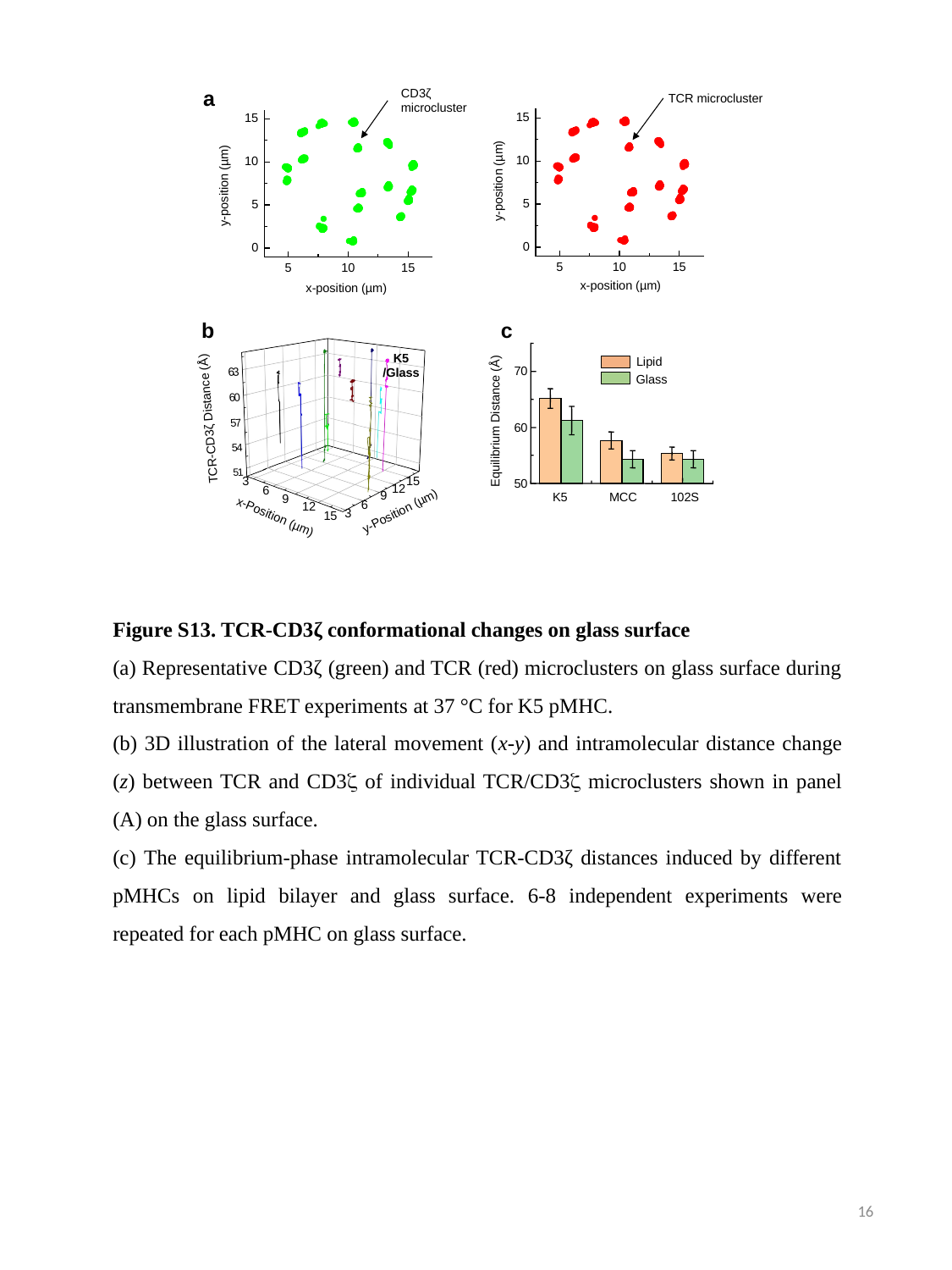

a
CD3ζ
microcluster
y-position (µm)
x-position (µm)
TCR microcluster
y-position (µm)
x-position (µm)
c
Lipid
Glass
Equilibrium Distance (Å)
70
60
50
K5
MCC
102S
b
d
K5
/Glass
TCR-CD3ζ Distance (Å)
15
3
12
6
9
9
6
12
y-Position (µm)
3
15
x-Position (µm)
Figure S13. TCR-CD3ζ conformational changes on glass surface
(a) Representative CD3ζ (green) and TCR (red) microclusters on glass surface during transmembrane FRET experiments at 37 °C for K5 pMHC.
(b) 3D illustration of the lateral movement (x-y) and intramolecular distance change (z) between TCR and CD3 of individual TCR/CD3 microclusters shown in panel (A) on the glass surface.
(c) The equilibrium-phase intramolecular TCR-CD3ζ distances induced by different pMHCs on lipid bilayer and glass surface. 6-8 independent experiments were repeated for each pMHC on glass surface.
16

## Slide 17
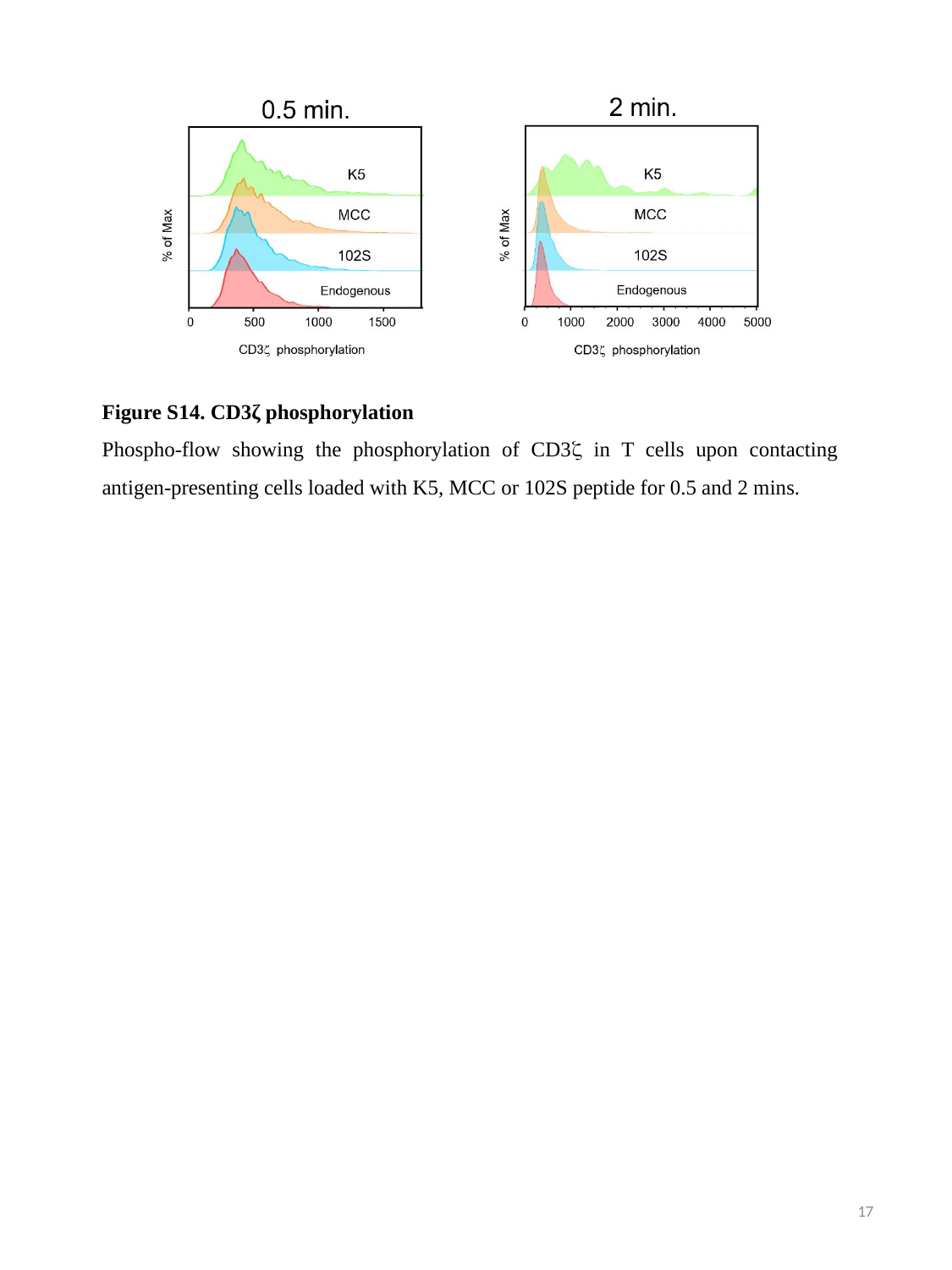

Figure S14. CD3ζ phosphorylation
Phospho-flow showing the phosphorylation of CD3 in T cells upon contacting antigen-presenting cells loaded with K5, MCC or 102S peptide for 0.5 and 2 mins.
17

## Slide 18
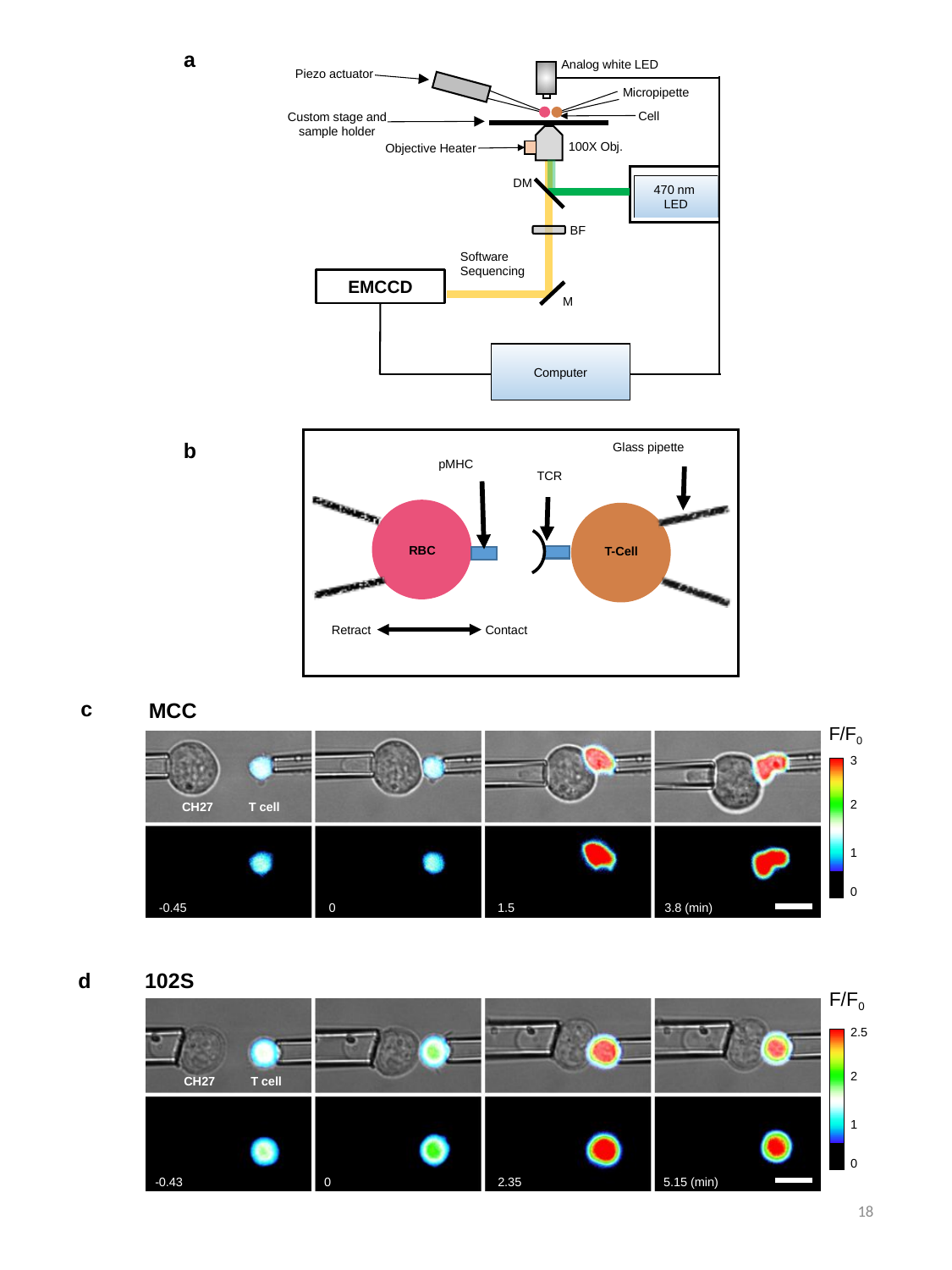

a
Analog white LED
Piezo actuator
Micropipette
Cell
Custom stage and sample holder
100X Obj.
Objective Heater
DM
470 nm
LED
BF
Software
Sequencing
EMCCD
M
Computer
Glass pipette
pMHC
TCR
RBC
T-Cell
Retract
Contact
b
c
MCC
F/F0
3
2
1
0
CH27
T cell
-0.45
0
1.5
3.8 (min)
d
102S
F/F0
2.5
2
1
0
-0.43
0
2.35
5.15 (min)
CH27
T cell
18

## Slide 19
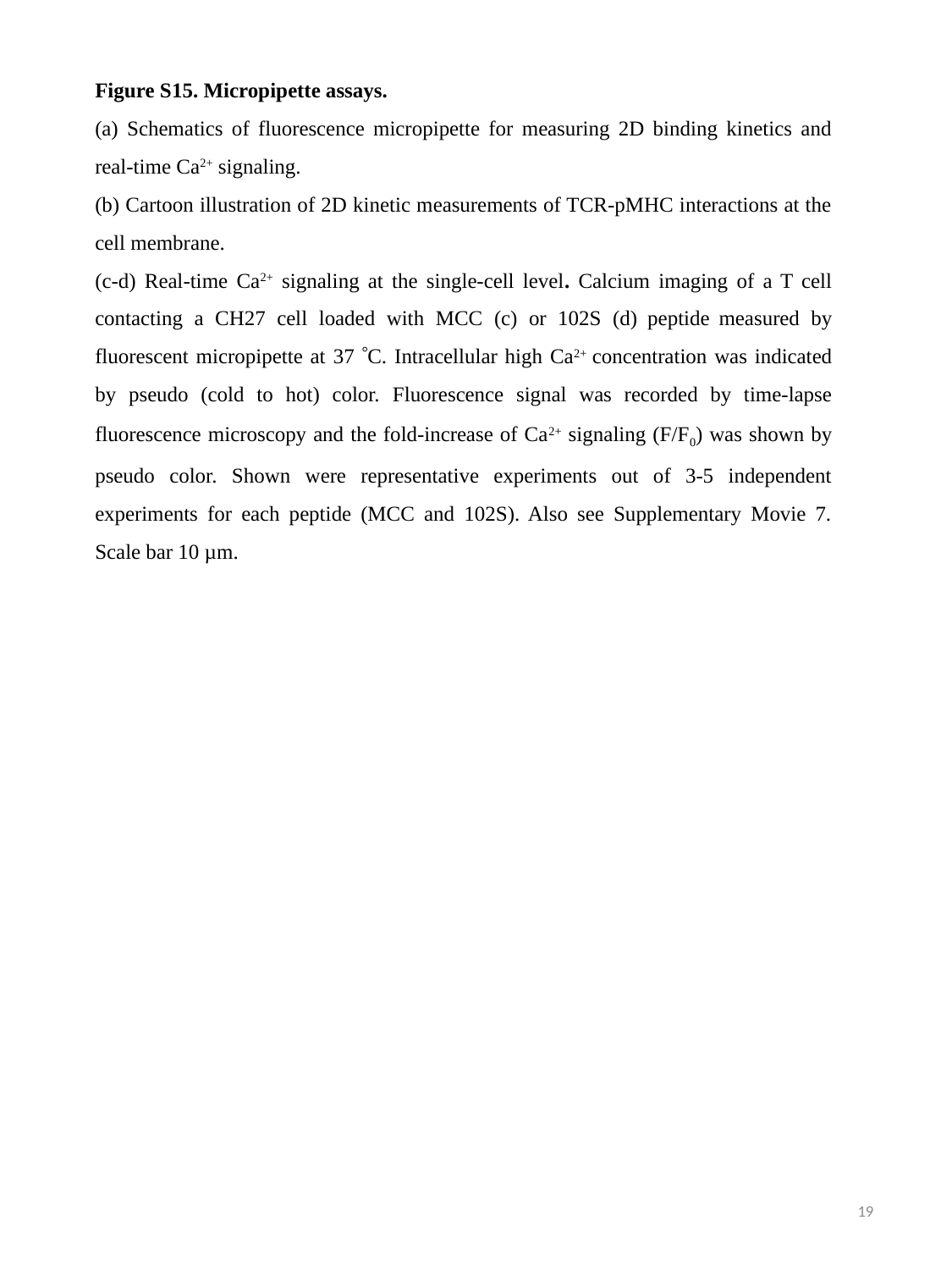

Figure S15. Micropipette assays.
(a) Schematics of fluorescence micropipette for measuring 2D binding kinetics and real-time Ca2+ signaling.
(b) Cartoon illustration of 2D kinetic measurements of TCR-pMHC interactions at the cell membrane.
(c-d) Real-time Ca2+ signaling at the single-cell level. Calcium imaging of a T cell contacting a CH27 cell loaded with MCC (c) or 102S (d) peptide measured by fluorescent micropipette at 37 °C. Intracellular high Ca2+ concentration was indicated by pseudo (cold to hot) color. Fluorescence signal was recorded by time-lapse fluorescence microscopy and the fold-increase of Ca2+ signaling (F/F0) was shown by pseudo color. Shown were representative experiments out of 3-5 independent experiments for each peptide (MCC and 102S). Also see Supplementary Movie 7. Scale bar 10 µm.
19

## Slide 20
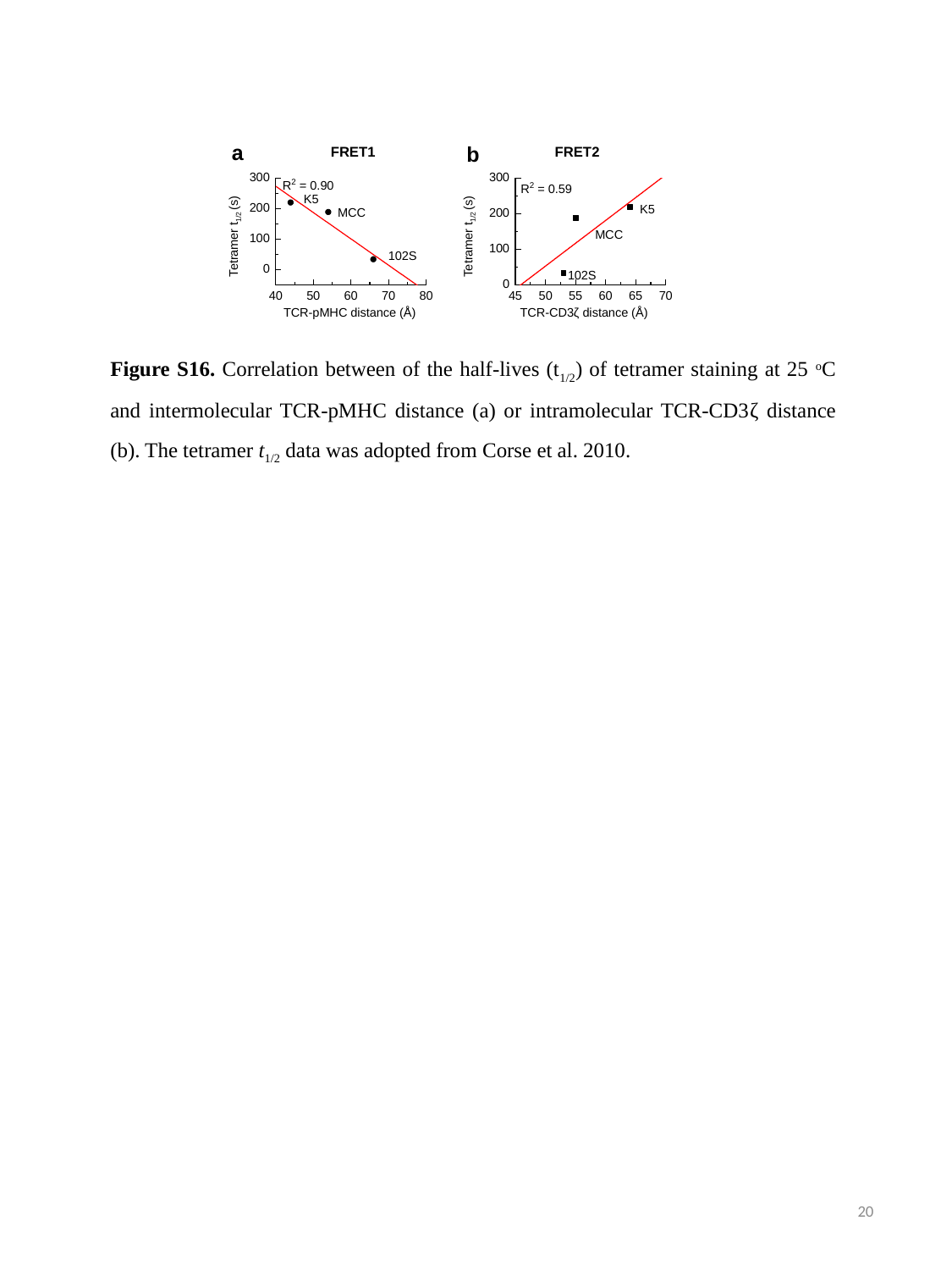

a
b
FRET1
FRET2
K5
Tetramer t1/2 (s)
Tetramer t1/2 (s)
K5
MCC
MCC
102S
102S
TCR-pMHC distance (Å)
TCR-CD3ζ distance (Å)
Figure S16. Correlation between of the half-lives (t1/2) of tetramer staining at 25 oC and intermolecular TCR-pMHC distance (a) or intramolecular TCR-CD3ζ distance (b). The tetramer t1/2 data was adopted from Corse et al. 2010.
20

## Slide 21
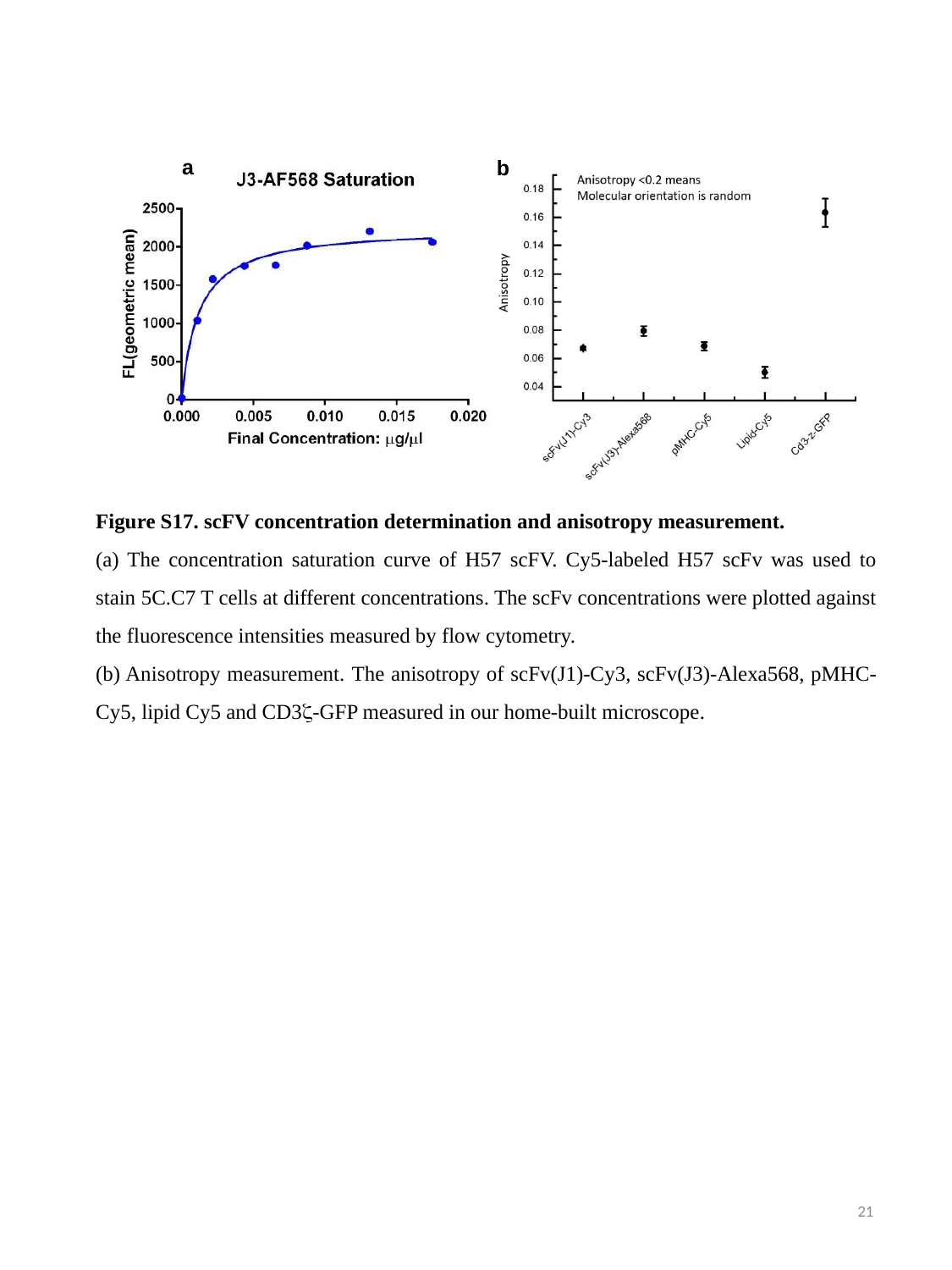

a
b
Figure S17. scFV concentration determination and anisotropy measurement.
(a) The concentration saturation curve of H57 scFV. Cy5-labeled H57 scFv was used to stain 5C.C7 T cells at different concentrations. The scFv concentrations were plotted against the fluorescence intensities measured by flow cytometry.
(b) Anisotropy measurement. The anisotropy of scFv(J1)-Cy3, scFv(J3)-Alexa568, pMHC-Cy5, lipid Cy5 and CD3-GFP measured in our home-built microscope.
21

## Slide 22
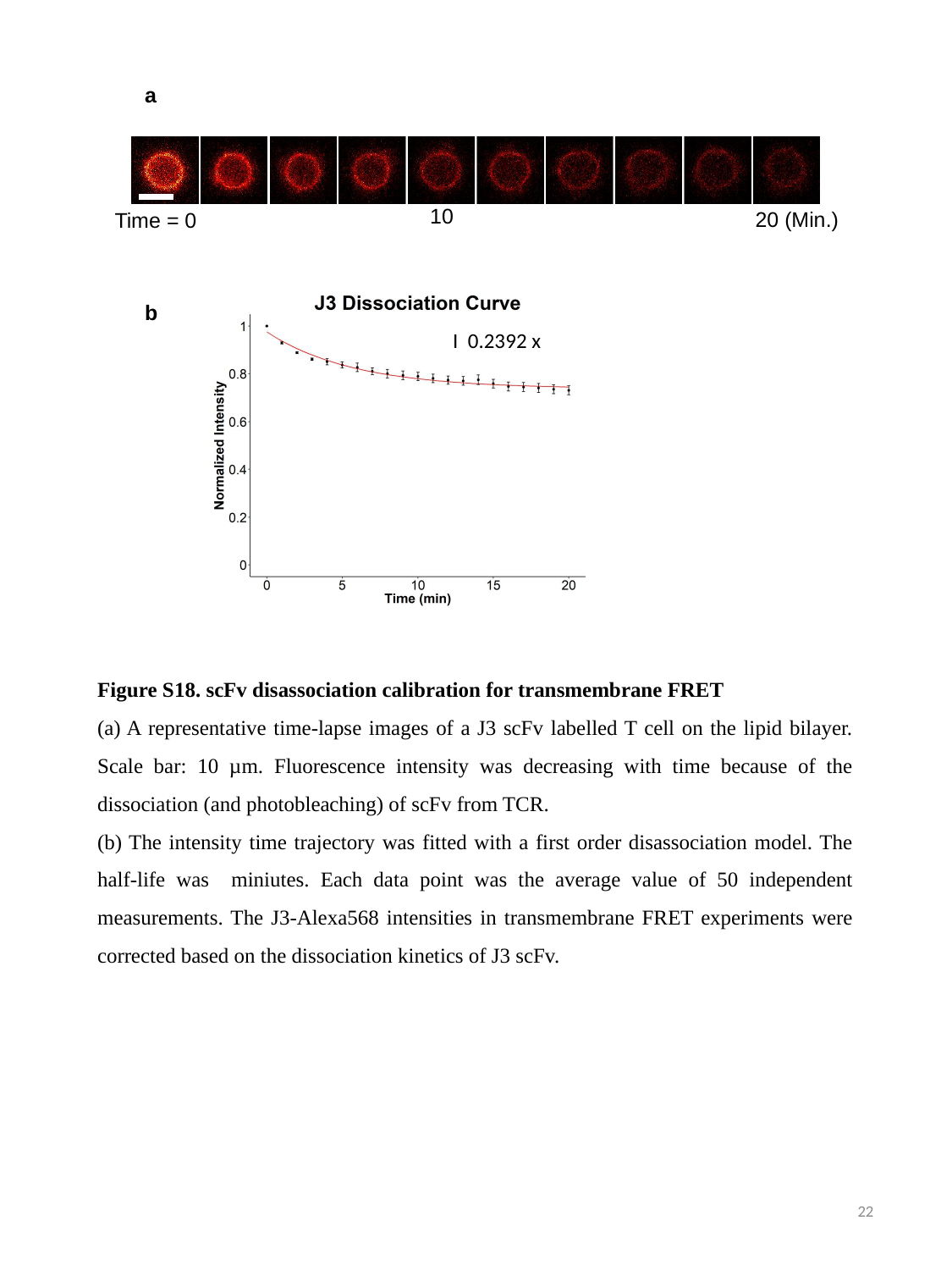

a
10
20 (Min.)
Time = 0
b
22

## Slide 23
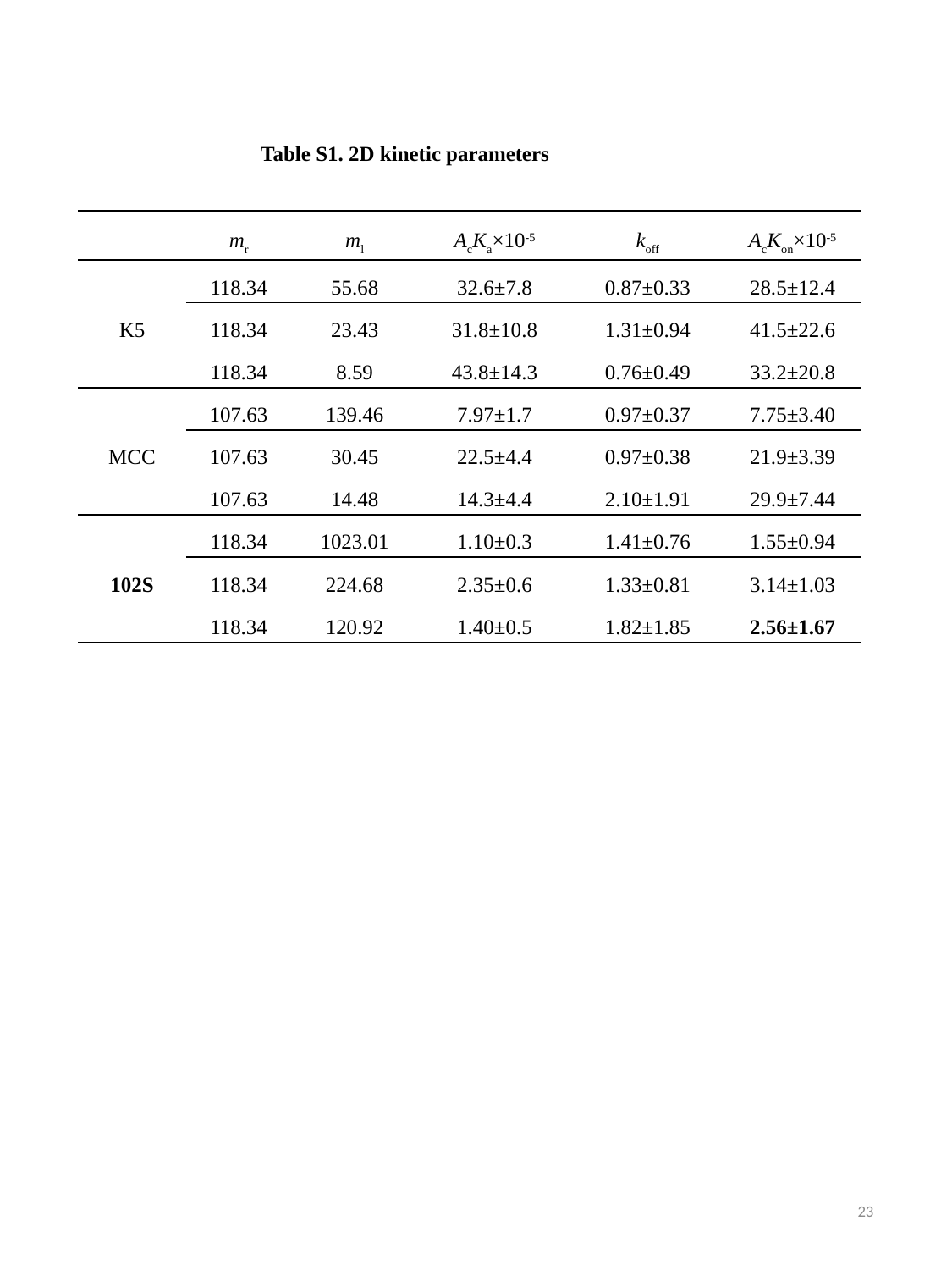

Table S1. 2D kinetic parameters
| | mr | ml | AcKa×10-5 | koff | AcKon×10-5 |
| --- | --- | --- | --- | --- | --- |
| K5 | 118.34 | 55.68 | 32.6±7.8 | 0.87±0.33 | 28.5±12.4 |
| | 118.34 | 23.43 | 31.8±10.8 | 1.31±0.94 | 41.5±22.6 |
| | 118.34 | 8.59 | 43.8±14.3 | 0.76±0.49 | 33.2±20.8 |
| MCC | 107.63 | 139.46 | 7.97±1.7 | 0.97±0.37 | 7.75±3.40 |
| | 107.63 | 30.45 | 22.5±4.4 | 0.97±0.38 | 21.9±3.39 |
| | 107.63 | 14.48 | 14.3±4.4 | 2.10±1.91 | 29.9±7.44 |
| 102S | 118.34 | 1023.01 | 1.10±0.3 | 1.41±0.76 | 1.55±0.94 |
| | 118.34 | 224.68 | 2.35±0.6 | 1.33±0.81 | 3.14±1.03 |
| | 118.34 | 120.92 | 1.40±0.5 | 1.82±1.85 | 2.56±1.67 |
23
